# Synthesis, Characterization, and Application of an Ecofriendly C/TiO_2_ Composite to Efficiently Remove Reactive Black 5 (RB-5) Textile Dye from Aqueous Solutions

**DOI:** 10.1021/acsomega.4c10884

**Published:** 2025-03-18

**Authors:** Lucas Destefani Paquini, Lília Togneri Marconsini, Bruno Sanches de Lima, Luciene Paula Roberto Profeti, Josimar Ribeiro, Demetrius Profeti

**Affiliations:** † Laboratório de Pesquisa e Desenvolvimento em Eletroquímica (LPDE), Universidade Federal do Espírito Santo, Campus Goiabeiras, Av. Fernando Ferrari, 29075-910 Vitória, Espírito Santo, Brazil; ‡ Programa de Pós-Graduação em Agroquímica, Universidade Federal do Espírito Santo, Alto Universitário, s/n., 29500-000 Alegre, Espírito Santo, Brazil; § Instituto de Física Gleb Wataghin, Universidade Estadual de Campinas—UNICAMP, 13083-859 Campinas, Espírito Santo, Brazil

## Abstract

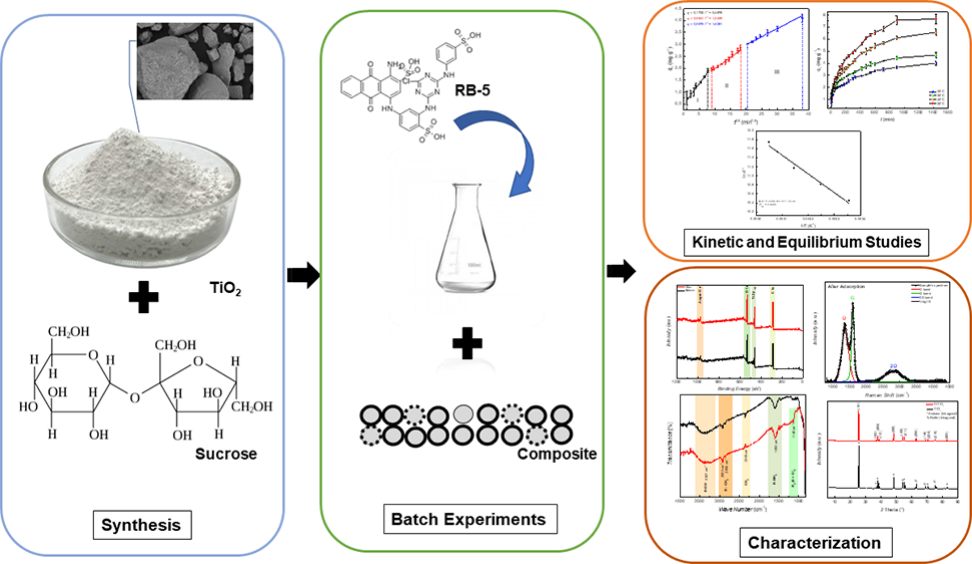

The textile industry is known for its high water consumption and production of toxic effluents, including azo dyes such as Reactive Black 5 (RB-5), which are resistant to removal. Adsorption offers a promising, cost-effective solution, particularly with value-added composites made from abundant materials. This study synthesized, characterized, and applied a C/TiO_2_-based composite to remove RB-5 from water. XRD analysis only confirmed anatase as the primary support, while FTIR detected adsorbate molecules on the C/TiO_2_ surface, marked by the appearance of a sulfone group band. Raman and XPS analyses indicated reduced Sp^2^ carbon content and lower graphitization after adsorption, probably due to mechanical stress. Additionally, nitrogen physisorption analysis demonstrated that the material is mesoporous, with a surface area of 56.24 m^2^ g^–1^, and a pore diameter of 9.41 nm. The composite exhibited strong affinity for anionic species like RB-5, especially at pH values below the point of zero charge (7.47). Batch studies demonstrated Avrami kinetic adsorption at a rate of 0.3023 min^–1^ (at 25 °C), while temperature effects followed the Arrhenius model, with an activation energy of +41.10 kJ mol^–1^. Sips isotherm data indicated a maximum adsorption capacity of 17.48 mg g^–1^ at 55 °C. Thermodynamic analysis confirmed an entropically controlled, endothermic, spontaneous process at high temperatures. These aspects confirm the potential of eco-friendly C/TiO_2_ composite for effective RB-5 removal from aqueous solutions.

## 1 Introduction

The development of the textile sector has significantly furthered global industrialization and the progress of modern societies.^1,2^ However, its mode of operation includes a series of characteristics that directly impact collective well-being and the environment. The degradation of water resources due to this industrial sector has become a striking factor in recent decades, mainly due to the frequent release of untreated wastewater containing heavy loads of highly toxic contaminants.^3−5^ Persistent organic pollutants (POP) are considered one of the most problematic species of these effluents, and current laws barely regulate these recalcitrant species.^6^ The main reason for this limitation lies in their ecotoxicological and behavioral effects, which the scientific community is yet to fully explain since the international monitoring program ignores a large portion of POP, hindering research and scientific dissemination on the subject.^7,8^

Within the wide range of POP products, dyes and pesticides emerge as the biggest concern for human health and equilibrium of the entire ecosystem.^9,10^ Dyes are chemical species with chromophore groups, conjugated unsaturated systems, and certain auxochrome groups in their structure that intensify their visual color effect due to their electron acceptor or donor systems.^11,12^ The most well-known chromophore groups include –C=C–, –C=N–, –C=O–, –N=N–, and –NO_2_, which change the coloration of these species in aqueous media, mainly due to their synergistic resonance processes.^13^

Dyes that fail to adhere to fabric in the textile transformation process are transformed into a liquid effluent with high chemical and biological oxygen demands. According to Rani et al.,^14^ the European Union stands out as the largest global producer of POP, with a significant annual volume of around 400,000,000 tons of pesticides and an impressive 1.6 billion tons of dyes. Additionally, according to the United States Environmental Protection Agency, the amounts of basic and nonfixed reactive dyes that can be discharged into an effluent total and 50–60%, respectively.

Among the POP classes described in the literature, azo dyes are one of the oldest and most common in the industry, being used to dye various materials. These species have a wide spectral range of colors, representing about 65% of commercially available dyes. According to Ramsay and Nguyen,^15,16^ azo dyes contain one or more azo groups (–N=N–), sulfone reactive groups [R–S(=O)_2_], and bonds between two or more aromatic rings, guaranteeing high structural stability due to resonance phenomena.^17^ Reactive Black 5 (RB-5) is one of the most modern and widely applied dyes in the azo class. It represents the main raw material for dyeing jeans, cotton, and polyester, besides being used in applications in biopharmaceutical analyses as an albumin inhibitor and in enzymatic saccharide metabolism.^18^ This azo dye has an azo group (–N=N–) and additional reactive groups, such as −SO_3_^2–^, –NH_2_, and –OH, which contribute to the high affinity of the molecule to textile fibers and its excellent attachment properties. RB-5 also contains aromatic rings with electrons that tend to energetically participate in oxidative electrochemical reactions, as per Da Costa Soares et al.^19^ and Yadav et al.^20^

Due to its high-water solubility, the color of RB-5 can change, and its turbidity may increase at low concentrations, triggering eutrophication processes in aquatic ecosystems.^18,21^ Considering this problem, several methodologies to treat azo-rich effluents have been proposed, especially precipitation, followed by coagulation, membrane filtration, ozonation, photocatalysis, advanced oxidative processes, advanced oxidative electrochemical processes, and adsorption,^22^ with the latter offering a promising alternative among the conventional methods to treat wastewater due to its excellent cost/efficiency indicators.^23^ Several materials have been used to remove or minimize the presence of various pollutants in aqueous solutions, including, for example, adsorbents derived from natural resources such as agricultural byproducts, which are sustainable, low-cost materials to remove dyes.^20,24−26^ Conventional adsorbents, such as active carbon, are still widely used, and modification or functionalization by biopolymeric derivatives may improve their removal parameters.^22^ Moreover, abundant and easily obtainable species may provide the key to the development of hybrid materials with higher added value than their isolated precursors, since composites/hybrids have been reported in a wide range of applications, including photocatalysis, water treatment, and antimicrobial properties.^27,28^

Composite materials can be prepared by combining organic–organic (e.g., chitosan-alginate, chitosan-protein, and chitosan-starch), organic–inorganic (e.g., chitosan-TiO_2_, TiO_2_-alginate, and starch-TiO_2_), and inorganic–inorganic hybrid substrates (e.g., TiO_2_–ZnO, TiO_2_–MgO, and TiO_2_–Ag).^29−32^ Several methodologies can synthesize these materials, including polymeric intercalation; sol–gel, hydrothermal, electrodeposition; chemical and physical vapor deposition; suspension; liquid phase deposition; and pyrolysis/copyrolysis.^33^ These methods effectively improve the chemical and mechanical properties of each component and provide new functionalities to the resulting combination.^34^ According to Anaya-Esparza et al.,^35^ one of the most reported precursors in the literature—titanium dioxide (TiO_2_)—is a versatile and chemically inert material with many relevant applications (food, pharmaceutical, biomedical, antimicrobial agent, environmental, and clean energy).^36−38^

The widespread use of TiO_2_ can be attributed to its exceptional physicochemical, mechanical, and photocatalytic properties,^39^ in addition to its reactivity, thermal stability, low cost, safety, biocompatibility, and ability to dope with other inorganic compounds.^40,41^ Although the main drawback of nano-TiO_2_ is its high agglomeration capacity, according to Guo et al.,^42^ associating biopolymers or macromolecules to its structure (starch, gums, chitosan, or saccharides) can help reduce spontaneous TiO_2_ agglomeration, improving the functional properties and removal of final composites.^43,44^ Regarding the preparation and application of composites in adsorption, materials based on carbon-SiO_2_ and carbon–TiO_2_ (C/TiO_2_) have received a great deal of attention recently.^45,46^ According to Liu et al.,^47^ silicon, titanium, and magnesium oxides are known to enhance the stability of carbon structures and improve their adsorption capacity for pollutants through π–π electron donor–acceptor interactions and pore-filling effects. Similarly, the proposed C/TiO_2_-based composite demonstrates the formation of structures with high affinity for pollutant removal while maintaining excellent structural stability during adsorption.^48,49^ In this context, the present study aims to synthesize and characterize a TiO_2_ composite integrated with a pyrolyzed carbon structure, specifically designed as an adsorbent for the removal of azo dyes, such as RB-5 textile dye, from synthetic effluent. Batch experiments were conducted to evaluate the kinetic and thermodynamic aspects of the adsorption process.

## 2 Materials and Methods

### 2.1 Synthesis of the C/TIO_2_ Composite

The composite was synthesized at a 0.1 C/TiO_2_ ratio across multiple batches. Initially, 2.100 g of sucrose were dissolved in 15 mL of ultrapure water at 120 °C. Subsequently, 5.000 g of titanium dioxide (TiO_2_) were added to the mixture, which was vigorously stirred to prevent the formation of TiO_2_ agglomerates. The resulting mixture was dried at 250 °C for 3 h and then subjected to pyrolysis at 600 °C for an additional 3 h in a furnace under an inert nitrogen (N_2_) atmosphere. The resulting samples were homogenized and segregated by particle size. Finally, adsorption assays were conducted to evaluate the material’s efficiency in removing RB-5 dye from aqueous solutions. The carbon content in the C/TiO_2_ coating was determined experimentally through gravimetric analysis in a muffle furnace. The analysis involved measuring the mass difference of a composite sample (∼0.1000 g) before and after calcination. The process was conducted at 500 °C for 60 min in air.

### 2.2 Characterization of the C/TIO_2_ Composite

X-ray diffraction (XRD) of the composite was carried out using the powder method in a Rigaku diffractometer (Mini-Flex 600 model). The peaks were collected at a 2θ range from 5 to 90°, with increments of 0.50° 2θ s^–1^ and a Cu Kα radiation source (λ = 1.541 Å).

Fourier transform infrared spectroscopy (FTIR) was carried out to obtain qualitative insights into the main functional groups present on the surface of the composite. The analysis was conducted using the attenuated total reflectance (ATR) method with a Deuterated l-alanine Doped Triglycine Sulfate (DLaTS) detector using a Bruker Tensor—27 system. Measurements were conducted over a wavelength range of 4000 to 600 cm^–1^, with 32 scan cycles per analysis at a sensitivity of 4 cm^–1^.

The scanning electron microscopy (SEM) analysis coupled with energy dispersive spectroscopy (EDS) was conducted using a scanning electron microscope (JEOL, model JSM-6010LA), with the filament operating at a voltage of 20.0 kV and a power of 1500 W. Prior to analysis, the C/TiO_2_ samples underwent metallization through gold sputtering (MED020 Sputter Coater—Baltec) to improve their electrical conductivity. Micrographs were subsequently acquired at magnifications of 1000× and 2500×, both before and after the RB-5 adsorption process.

The C/TiO_2_ composite was structurally characterized using Raman spectroscopy and X-ray photoelectron spectroscopy (XPS), both before and after adsorption of the Reactive Black 5 (RB-5) azo-dye, at an initial concentration of 100 mg L^–1^, over 24 h at 25 °C. Raman spectra for the material were obtained using a Witec microscope equipped with Nikon lenses and a CCD-type Peltier cell. The samples were excited by an Ar-ion laser operating at a wavelength of 514 nm (green laser). Data processing was carried out using a Voigt fit with specialized software, and the results are presented in graphs and tables.

The chemical states and surface transitions of the composite were examined using XPS. The data were acquired using a Scientia Omicron ESCA spectrophotometer with a monochromatic X-ray source (Al Kα = 1486.7 eV), operating at 280 W and a constant energy step of 50 eV. The XPS peaks were characterized by fitting the spectral data to the Gaussian–Lorentzian function, with Shirley background. During the fitting process, the full width at half maximum (fwhm) values were fixed to the C 1s component, while peak positions were allowed to vary by up to 0.5 eV within the binding energy range.

Area and porosity analysis using N_2_ physisorption were also performed in this study. Initially, ampules with about 0.2000 g of the adsorbent were weighed to analyze the area and determine the porosity of the C/TiO_2_ composite. The samples were then degassed by subjecting them to low pressure/vacuum (0.035 kPa) under heating at a ramp of 10 °C min^–1^ before fixation in isothermal conditions (200 °C) for 30 min. Surface area and porosity analyses were then conducted through N_2_ physisorption at a low temperature (77.2 K), applying mesopore analysis under a 0.01–0.99 p/p° range for an adjusted 4–5 h analysis time. Acquiring and processing data included applying the Brunauer, Emmet, and Teller (BET); Langmuir, Barrett, Joyner, and Halenda; and the Harkins and Jura t-plot adsorption models. These steps (degassing/analysis) were conducted in a JWGB Meso 112 area and porosity analyzer.

The pH at point of zero charge (pH_PZC_) was determined by preparing a system with 0.1000 g of the composite in a 10.00 mL NaCl (0.1 mol L^–1^) aqueous solution under 11 initial pH conditions (2.0–12.0) adjusted with HCl (0.1 mol L^–1^) and/or NaOH (0.1 mol L^–1^) solutions. In this experiment, the NaCl solution was applied to control the ionic strength of the medium and promote a greater constancy of solution activity coefficients across pH values. The system (adsorbent + NaCl solution) was left to rest for 1440 min before the final pH was measured with a Quimis pH-meter (Q400MT) for subsequent data processing.

### 2.3 Initial Tests

The study of the effect of the initial pH on the adsorption process was carried out by varying the pH from 2.0 to 12.0 using 0.01 mol L^–1^ HCl and NaOH solutions to adjust the pH. An adsorptive system was then prepared with about 0.1000 g of the C/TiO_2_ composite and 10.00 mL of the RB-5 solution at an initial concentration of 50 mg L^–1^. The system was stirred at 100 rpm using an orbital shaker with temperature control at 25 °C for 1440 min. The solutions were then filtered to separate the suspended solid from the remaining solution. The filtered solutions were analyzed using a UV–vis absorption spectrophotometer (Thermo-Fischer Scientific GENESYS 10S) at a 599 nm wavelength, the maximum absorption peak of the dye.

To evaluate the effect of the initial concentration on adsorption, about 0.1000 g of the composite was weighed in 125 mL Erlenmeyer flasks. Subsequently, 10.00 mL of the RB-5 solution were added to each Erlenmeyer along with the adsorbent at concentrations ranging from 50 to 300 mg L^–1^ at an initial pH of 5.5 at 25 °C. The adsorbate + adsorbent system was placed in an orbital shaker at 100 rpm for 24 h. The samples were then filtered and analyzed in a UV–vis absorption spectrophotometer at a 599 nm wavelength. Analyses showed a specific absorbance value that was converted into concentration values using the calibration curve method. The percentage of dye removed by the composite, RE (%), was then validated following eq 1
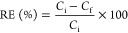
1

On the other hand, loading (*q*), given by the amount in milligrams of dye adsorbed per 1 g of adsorbent, is shown in eq 2
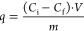
2where *C*_i_ refers to the initial concentration (mg L^–1^) of the dye; *C*_f_ to the final concentration of the solution; *V* to the volume of the added dye aliquot (L); and *m* to the composite mass in the system (g).

In addition, experiments were conducted to assess the impact of composite formation on dye adsorption by comparing the removal performance of C/TiO_2_ with its individual components, C and TiO_2_. Furthermore, experiments were also performed to evaluate the effect of carbon content in the coating, with the aim of identifying the optimal carbon composition for dye removal. Thus, batch experiments were carried out using 0.1000 g of adsorbent (C, TiO_2_ and C/TiO_2_ at 5% C, 10% C, 15% C, 20% C, 30% C, and 50% C) placed in 125 mL Erlenmeyer flasks. Subsequently, a 10.00 mL volume of RB-5 dye solution (100 mg L^–1^) was added to the flasks containing the preweighed adsorbents. The adsorbate + adsorbent system was then placed in an orbital shaker at 100 rpm for 180 min at 25 °C. The samples were then filtered and analyzed using a UV–vis absorption spectrophotometer at a wavelength of 599 nm and removal and loading were calculated and subsequently plotted.

### 2.4 Adsorption Kinetics

To carry out the adsorption kinetics experiments, adsorptive systems were set up with 0.1000 g of the C/TiO_2_ composite, weighed in 125 mL Erlenmeyer flasks, with the subsequent addition of 10.00 mL of the RB-5 solution at a fixed concentration of 100 mg L^–1^. The system (dye + adsorbent), totaling 10 g L^–1^, was then placed in a Lucadema orbital shaker (luca-223) at 100 rpm cycles at varying temperature conditions (from 25 to 55 °C). Considering the kinetic nature of this evaluation, the samples were removed from the shaker at predetermined time intervals (1–1440 min) and filtered and analyzed using a UV–vis absorption spectrophotometer at a fixed 599 nm wavelength.

All kinetic adsorption behavior experiments were performed in duplicate. The amount of RB-5 adsorbed per unit mass of composite at instant *t* was measured using eq 3
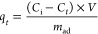
3where *q*_*t*_ corresponds to the amount of dye adsorbed per unit mass of the composite (mg g^–1^) at instant *t*; *C*_i_ and *C*_*t*_ to dye concentration (mg L^–1^) at the initial moment and at instant *t*, respectively; and *m*_ad_ to the mass (g) of the composite in the adsorption tests.

### 2.5 Adsorption Equilibria

For the adsorption equilibrium experiments, C/TiO_2_ adsorptive systems weighing 0.1000 g were set up in 125 mL Erlenmeyer flasks, to which 10.00 mL of the RB-5 solution were added. As this investigation is related to adsorption equilibrium, a concentration range of 10–300 mg L^–1^ was adopted. This 10 g L^–1^ system (dye + adsorbent) was then placed in a 100 rpm cycle orbital shaker at temperatures varying from 25 to 55 °C. To ensure full equilibrium, the system was agitated for 1440 min. The samples were then filtered and analyzed using a UV–vis absorption spectrophotometer at a fixed wavelength of 599 nm.

Equilibrium was subsequently analyzed through the relationship between the amount of dye adsorbed onto the composite (*q*_e_) and the equilibrium concentration (*C*_e_). The amount adsorbed per unit mass of composite was evaluated using eq 4
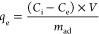
4where *q*_e_ corresponds to the amount of dye adsorbed per unit mass of the composite (mg g^–1^); *C*_i_ and *C*_e_ to the initial and final dye concentration (mg L^–1^), respectively; and *m*_ad_ to the mass (g) of the composite used in the adsorption tests.

### 2.6 Data Processing

Adsorbance values were converted into concentration measurements using a calibration curve. The experimental equilibrium data were then fitted to nonlinear models, including Langmuir, Freundlich, Redlich–Peterson, Sips, Liu, Khan, and Temkin isotherms. The thermodynamic state functions of enthalpy (Δ*H*°) and entropy (Δ*S*°) were determined using the linear Van’t Hoff model, while Gibbs free energy (Δ*G*°) was calculated using the general Gibbs free energy equation.

The adsorption kinetics data from the replicates were fitted to nonlinear models, including the pseudo-first order (PFO), pseudo-second order (PSO), Elovich, and Avrami models. Additionally, the kinetic data revealed the mass transfer behavior of the adsorption process using the Weber–Morris multilinear model. Finally, the experimental replicate data were used to estimate the apparent activation energy of the adsorption process through Arrhenius’ empirical model.

The complete list of theoretical-mathematical models in the adsorption evaluation is shown in Tables 1 and 2 below. Table 1 shows the kinetic models used in the adsorption assessment, including the nonlinear empirical kinetic models and the Weber–Morris and Arrhenius’ linear models.

**Table 1:** Mathematical Models to Assess the Adsorption Kinetics of RB-5 on the C/TiO_2_ Composite

model	equation	parameters
PFO^a^	*q*_*t*_ = *q*_*e*_(1-*e*^–*k*^_1_^*t*^)	*q*_*t*_: adsorption capacity at *t* (mg g^–1^)
		*q*_e_: equilibrium adsorption capacity (mg g^–1^)
		*k*_1_: pseudo-first order rate constant (min^–1^)
PSO^a^	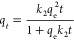	*q*_*t*_: adsorption capacity at *t* (mg g^–1^)
		*q*_e_: equilibrium adsorption capacity (mg g^–1^)
		*k*_2_: pseudo-second order rate constant (g mg^–1^ min^–1^)
Elovich^a^	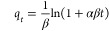	*q*_*t*_: adsorption capacity at *t* (mg g^–1^)
		α: initial adsorption rate (mg g^–1^ min^–1^)
		β: desorption constant (g mg^–1^)
Avrami^a^	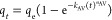	*q*_*t*_: adsorption capacity at *t* (mg g^–1^)
		*q*_e_: equilibrium adsorption capacity (mg g^–1^)
		*k*_AV_: Avrami’s rate constant (min^–1^)
		*n*_AV_: fractional order
Weber and Morris^b^	*q*_*t*_ = *k*_d_*t*^0.5^ + *C*	*k*_d_: intraparticle diffusion constant (mg g^–1^ min^–^0.5)
		*C*: boundary layer thickness (mg g^–1^)
Arrhenius^b^	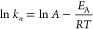	*k*_*n*_: kinetic constant
		*A*: pre-exponential factor
		*E*_A_: activation energy (J mol^–1^)

a Nonlinear mathematical model.

b Linear mathematical models, in which parametrization treatments are necessary. Source: The authors (2024), adapted from Lima, Adebayo and Machado,^50^ Ruthven^51^ and Rodrigues et al.^52^

**Table 2:** Mathematical Models Used to Assess the Adsorption Equilibrium of RB-5 on the C/TiO_2_ Composite

model	equation	parameters
Langmuir^a^	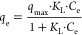	*K*_L_: Langmuir equilibrium constant (L mg^–1^), *q*_max_: maximum adsorption capacity (mg g^–1^)
		*C*_e_: equilibrium concentration (mg L^–1^)
Freundlich^a^	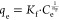	*K*_f_: Freundlich equilibrium constant (L mg^–1^), *n*_F_: degree of heterogeneity
		*C*_e_: equilibrium concentration (mg L^–1^)
Redlich–Peterson^a^	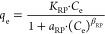	*K*_RP_: Redlich–Peterson equilibrium constant (L g^–1^), *a*_RP_: Redlich–Peterson constant (mg L^–1^)^−β^
		*C*_e_: equilibrium concentration (mg L^–1^)
		β_RP_: Redlich–Peterson exponent
Sips^a^	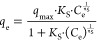	*K*_S_: Sips equilibrium constant (L mg^–1^), *q*_max_: maximum adsorption capacity (mg g^–1^)
		*C*_e_: equilibrium concentration (mg L^–1^), *n*_S_: Sips exponent
Liu^a^	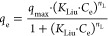	*K*_Liu_: Liu equilibrium constant (L mg^–1^), *q*_max_: maximum adsorption capacity (mg g^–1^)
		*C*_e_: equilibrium concentration (mg L^–1^), *n*_L_: Liu exponent
Khan^a^	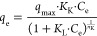	*K*_K_: Khan equilibrium concentration (L mg^–1^), *q*_max_: maximum adsorption capacity (mg g^–1^)
		*C*_e_: equilibrium concentration (mg L^–1^), *n*_K_: Khan exponent
Temkin^a^	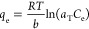	*a*_T_: Temkin isotherm constant (L mol^–1^)
		*b*: Temkin constant associated to adsorption enthalpy (L g^–1^)
		*C*_e_: equilibrium constant (mg L^–1^)
van’t Hoff^b^	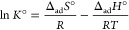	Δ_ad_*S*°: adsorption entropy (J K^–1^ mol^–1^)
		Δ_ad_*H*°: adsorption enthalpy (J mol^–1^)
		*R*: ideal gas constant (8314 J K^–1^ mol^–1^)
		*T*: temperature (K)
		*K*°: thermodynamic adsorption constant (adimensional)
thermodynamic adsorption constant	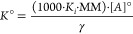	*K*°: thermodynamic adsorption constant (adimensional)
		γ: activity coefficient
		*K*_*i*_: isotherm best fit constant (L mg^–1^)
		MM: Molar mass of adsorbate (g mol^–1^)
		[*A*]°: Standard concentration (mol L^–1^)
Gibbs equation	Δ_ad_*G*° = −*RT* ln *K*°	Δ_ad_*G*°: adsorption free energy (J mol^–1^)
		*R*: ideal gas constant (8314 J K^–1^ mol^–1^)
		*T*: temperature (K)
		*K*°: thermodynamic adsorption constant (adimensional)

a Nonlinear mathematical model.

b Linear mathematical models, in which parametrization treatments are necessary. Source: The author (2024), adapted from Lima, Adebayo and Machado^50^ and Bazzarella et al.^53^

Table 2 presents all the models used for the adsorption equilibrium assessments.

The statistical analysis included the coefficients of determination (*R*^2^), the chi-squared test (χ^2^) for nonlinear regression, and standard deviations for the mean equilibrium and kinetics adsorption data. The regression models were implemented using C language software, which utilized the Levenberg–Marquardt algorithm for nonlinear regression cases.

## 3 Results and Discussion

### 3.1 Characterization

#### 3.1.1 X-ray Diffraction

**Figure 1:**
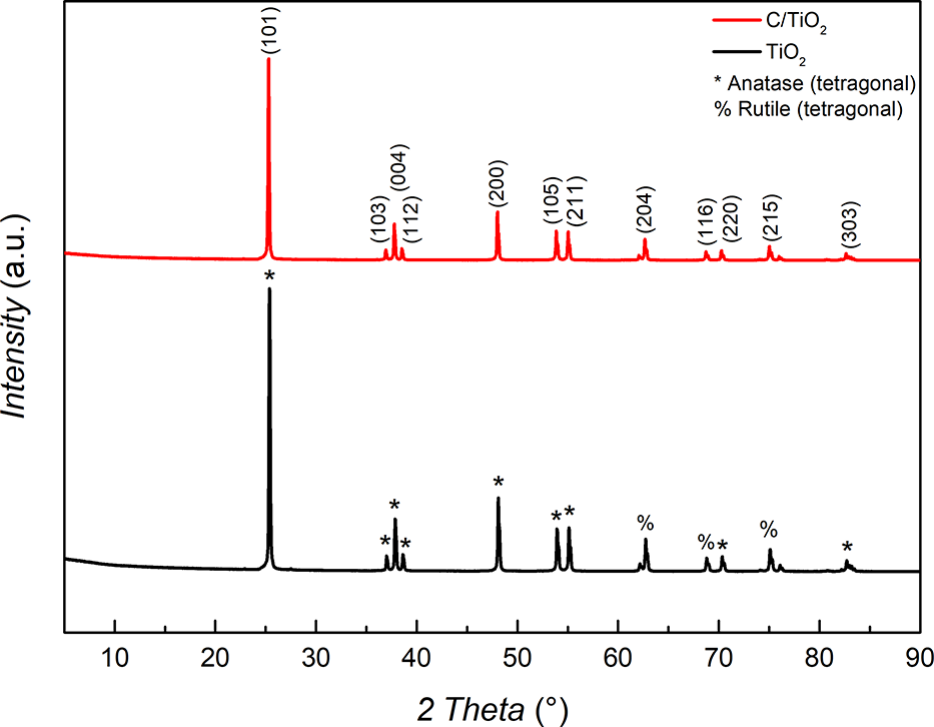
Profile related to overlapping X-ray diffraction patterns of (—) TiO_2_ and (—) C/TiO_2_. Experimental conditions: Δ°2θ = 5–90°, increments: 0.50 °2θ s^–1^, radiation: Cu Kα (λ = 1.541 Å).

Figure 1 shows the diffraction profile of the C/TiO_2_ composite before RB-5 adsorption and the isolated profile of the TiO_2_ support.

The sharp, narrow peaks in the diffraction profiles indicate that both samples exhibit high crystallinity. Moreover, the diffraction patterns of the samples showed minimal differences, suggesting that the C/TiO_2_ composite and the individual support are structurally similar.^54^ Another point of interest refers to the diffraction peaks. Following the JCPDS booklet linked to the ICDD PDF 2 database showed phases °2θ = 25.32, 36.96, 37.8, 38.56, 48.02, 53.88, 55.04, 68.74, 75.02, and 82.68° as characteristic of the anatase structure of TiO_2_ (PDF 2 ICDD database—00-004-0477). On the other hand, the phases at °2θ = 62.64, 68.69, and 75.11° (ICDD PDF 2 database—00-155-55) belong to the rutile structure of TiO_2_. This emphasizes that it was not possible to find considerable differences in the diffraction profiles of the substrate and the prepared composite as their diffraction peak intensities only varied slightly. This behavior may therefore stem from two factors: (*i*) the amorphous nature of the carbon coating; or (*ii*) the low concentration of carbon structures (graphite) on the TiO_2_ substrate surface. Moreover, potential amorphous peaks at 22.4 and 43.04 °2θ were related to the disordered structures of graphitic and hexagonal carbons and may have stemmed from obscured peaks due to the high intensities of the obtained crystalline profiles, thus making it impossible to identify them using a diffractogram.^55,56^

The estimation of the average crystalline size of the C/TiO_2_ composite showed that applying the Scherrer equation indicated a material with an average crystallite size (*D*) of ≈1077 nm. The estimate of this value was made for the phase at °2θ = 25.32°, referring to anatase (101).^56^

#### 3.1.2 Fourier Transform Infrared Spectroscopy Analysis

**Figure 2:**
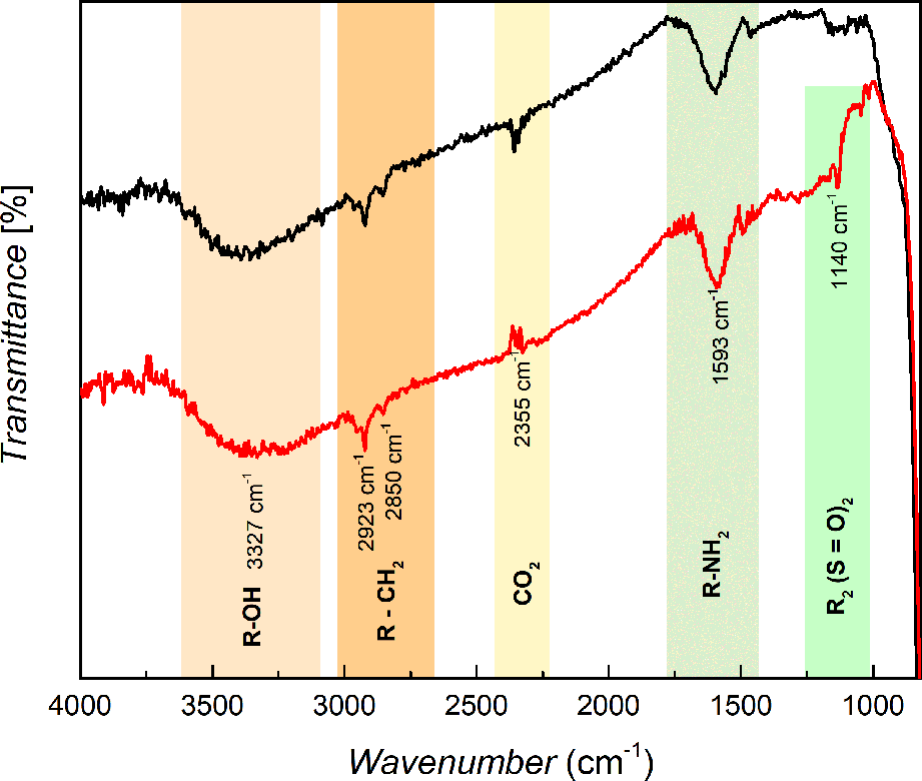
Spectral behavior obtained by the Fourier transform infrared spectroscopy with the C/TiO_2_ composite before and after the adsorption of the RB-5. Experimental conditions: method: attenuated total reflectance; wavelength: 4000–650 cm^–1^; cycles: 32; sensitivity: 4 cm^–1^.

Infrared absorption spectroscopy analysis provided crucial information regarding the vibrational profiles of the functional groups on the adsorbent surface.^57^ This qualitative technique can demonstrate the effect of certain procedures, such as dye adsorption, vibrational behavior, and presence and availability of certain functional groups.^58,59^Figure 2 shows the spectral behavior of the composite before and after adsorption.

The spectral profiles showed the main bands and the correlation between them and the potential functional groups on the surface of the adsorbent. In both spectra (C/TiO_2_ and C/TiO_2_ + RB-5), the absorption band at 3327 cm^–1^ was related to the stretching of the hydroxyl functional groups (−OH) in primary alcohols; whereas those at 2923 and at 2850 cm^–1^ showed two thin, low-intensity bands that corresponded to the vibrational profile of the stretching of aliphatic groups (−CH_2_) bonded to aromatic rings.^10^ The band at 2365 cm^–1^ refers to the stretching of the C=O bond in the CO_2_ molecules, which mainly occurred due to the environment in which the spectroscopic analysis was conducted.^59^ At 1539 cm^–1^, a medium-intensity band corresponding to angular deformation in the plane of the structures referred to the amine group (−NH_2_), and a slight increase in its intensity occurred after RB-5 adsorption onto the composite.^60^ Moreover, at 1140 cm^–1^, a thin, low-intensity band only emerged in the composite spectrum after adsorption, which is intrinsically related to the symmetrical angular deformation of sulfone groups [R_2_–S(=O)_2_].^10,60^

Correlating the spectra before and after adsorption shows that the spectrum of the material after adsorption differed slightly from the material prior to adsorption. Moreover, the sulfone group [R_2_–S(=O)_2_] in the postadsorption material can be associated with the structure of the adsorbed RB-5. In addition, a slight increase in the intensity of the amine group (−NH_2_) related band may also indicate the dye in the adsorbent structure.

Wamba et al.^59^ studied the synthesis of a 3-aminopropyltriethoxysilane-based composite to remove Bright Green 1 and Reactive Black 5. The authors performed Fourier transform infrared spectroscopy of the composite before and after adsorption and found groups belonging to the structure of the dyes on the surface of the material used as an adsorbent. Moreover, it was possible to confirm the presence of RB-5 on the surface of the composite, in part, from qualitative evidence on its spectral behavior.

#### 3.1.3 Scanning Electron Microscopy

The scanning electron microscopy (SEM) analysis enabled evaluation of the morphological characteristics of the composite both before and after adsorption. Figure S1 presents the micrographs corresponding to these two distinct conditions, at various magnifications. Based on the profiles presented, all the micrographs reveal a relatively rough surface characterized by the presence of irregular shapes, random arrangements, and varying grain sizes. In the materials before adsorption, thin clusters and finely deposited lamellae are observed on a more rigid and uniformly distributed structure, particularly at magnifications of 1000× and 2500×.^61^ Consistent with the findings of Song et al.,^48^ these clusters can be attributed to the amorphous carbon structures present in the C/TiO_2_ coating. Conversely, the micrographs obtained after adsorption show a significant loss of the fine layer of deposited clusters, more clearly exposing the support structures, which appear more regular and defined. Furthermore, variations in contrast intensity are observed in all the micrographs, which may be due to both the surface topography and the chemical nature of the material.^62^ Regarding the morphological changes observed in the composite before and after adsorption, it is anticipated that these changes are a direct consequence of the physical wear caused by the mechanical stress experienced by the material during batch adsorption experiments, particularly due to prolonged agitation. According to Xu et al.,^63^ mechanical wear, which can include abrasion, cracking, and plastic deformation of the composite surface, has the potential to alter the initial physicochemical properties of C/TiO_2_. As a direct consequence, the lifespan or number of cycles of the composite may be reduced, as the material’s efficiency as an adsorbent depends on its structural integrity and regeneration capacity.^64^ Although the carbon coating may initially enhance the composite’s adsorption capacity by increasing the active surface area and affinity for dye molecules, it can eventually contribute to reduced material durability and efficiency.^65^ Consequently, the loss of these structures decreases the availability of functional groups on the surface, which are essential for facilitating chemical adsorption processes. Overall, all the images reveal a rough surface, which provides a relatively large surface area for the composite.

Regarding the elemental analysis, energy dispersive X-ray spectroscopy (EDS) coupled with SEM was used to identify and quantify the primary elements composing the material. The results (Table S1) show the key elements constituting the C/TiO_2_ composite, along with their respective mass percentages (% wt) before and after the adsorption process. The data show that Ti and O were identified as the most abundant elements in the composite. This is because these elements form the support structure of C/TiO_2_, arranged in tetragonal configurations in the Anatase and Rutile phases.^66^ Additionally, the EDS analysis revealed significant variations in the mass percentages of these elements after the adsorption process. Initially, oxygen made up about 61.11% of the material’s mass, while Ti accounted for approximately 19.50%.^64^ After the batch process, there was a sharp increase in the Ti content, rising to 58.83% by mass, with a reduction in oxygen content to 20.33%. This increase in Ti can be corroborated by the behavior observed in the micrographs, where the physical wear caused by the adsorption experiments led to greater exposure of the support structure. Similarly, this effect also explains the slight reduction in the carbon content on the surface of the composite. The removal of surface carbon layers due to agitation during the adsorption tests contributed to a decrease in material carbon content of approximately 1.04%.^62^

#### 3.1.4 Spectroscopy Raman and X-ray Photoelectron Spectroscopy Analysis

**Figure 3:**
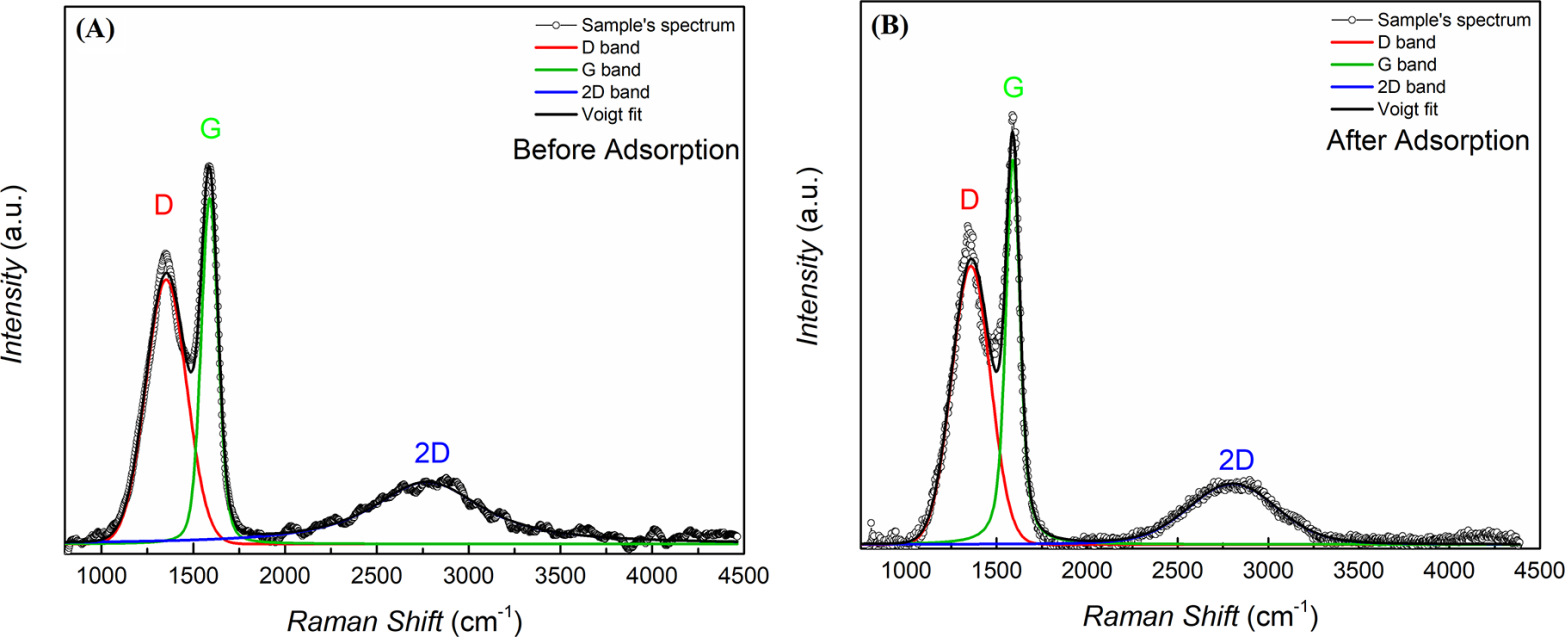
FT-Raman scanning spectra for the C/TiO_2_ composite under the following conditions: (A) before adsorption; and (B) after adsorption with RB-5. Experimental conditions: microscope: Witec; λ = 514 nm.

The Raman spectroscopy analyses aimed to further evaluate the structural characteristics of the C/TiO_2_ composite, both before and after RB-5 dye adsorption. Based on this premise, Figure 3 shows the scanning spectra of C/TiO_2_ and C/TiO_2_ + RB-5, and Table S2 presents the parameters derived from the spectral scanning of the composite. This table includes the intensities of the D and G bands, in addition to the graphitization index of the material, expressed as the ratio between the respective band intensities.

The spectral data reveal the prominent presence of two peaks at ∼1352.54 and ∼1589.68 cm^–1^, which can be attributed to the D and G bands of Sp^2^ carbon sites.^67^ These bands were identified through the nonlinear Voigt model regression of the spectral data. In both validated spectra, the presence of the D band indicates the occurrence of A_1g_ symmetry at the carbon sites, with potential structural disorder in the composite coating (amorphous carbon). On the other hand, the G band signifies the vibrational states of C–C bonds in Sp^2^ hybridization and E_2g_ symmetry.^68,69^

The intensity of the D band tends to be slightly higher after the adsorption process, compared to the G band, confirming that adsorption increased amorphous carbon content on the C/TiO_2_ surface.^68,70^ Accordingly, the G band intensity slightly decreased after adsorption, suggesting a disruption of the graphitic states in Sp^2^ hybridization and E_2g_ symmetry. These findings suggest that dye attachment to the C/TiO_2_ surface and the mechanical stress from the agitation process altered the organization of the carbon layer, especially after the experiments with RB-5.

Studies by Paz et al.^71^ reported that the adsorption of an azo dye mixture onto a metallic-organic-framework (MOF) led to changes in the C–C bond conformation and hybridization, making the surface locally more disordered. In addition, another notable feature in the spectra is the 2D band, observed around ∼2780–2800 cm^–1^, which, according to Zhang et al.,^72^ indicates a secondary vibration of the original D band and suggests the presence of disordered C–C and C=C structures. Finally, the ratio of D to G band intensities was examined (Table S2). This ratio remained close to 1.0 across all conditions, indicating a more amorphous than graphitic nature of the surface carbon structures.^68,73^

**Figure 4:**
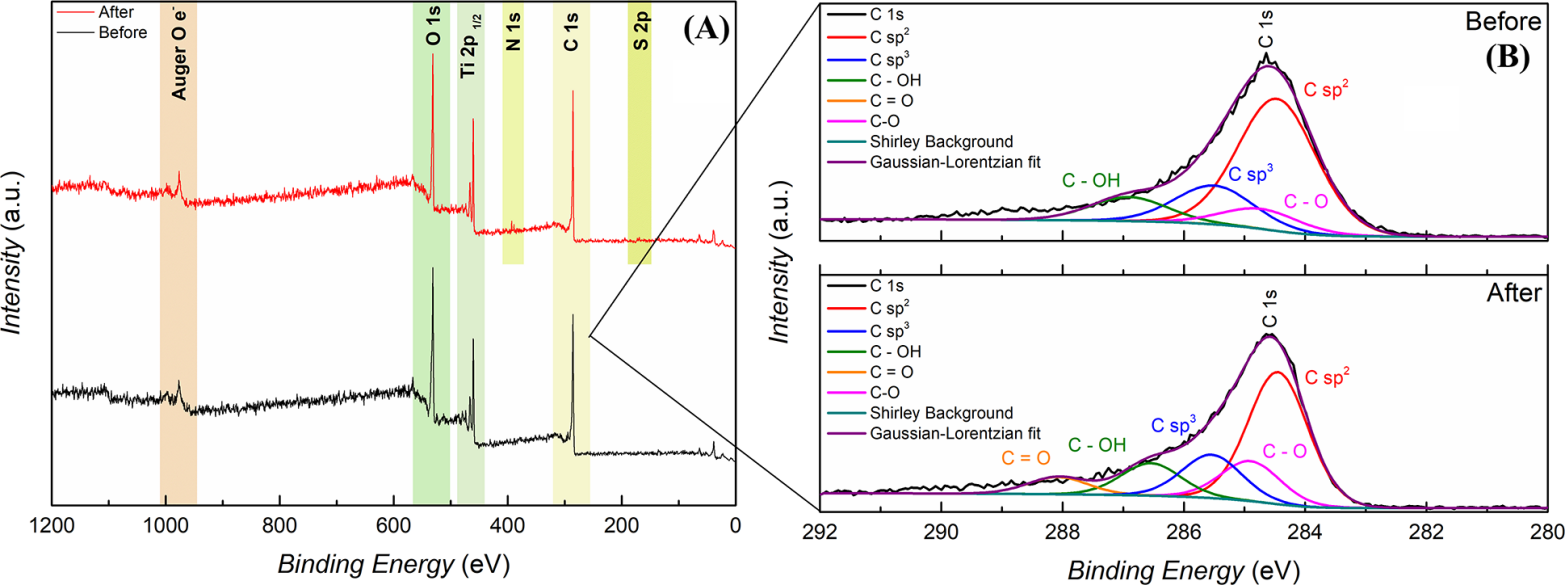
(A) XPS survey profile and (B) C1 s high-resolution spectra related to C/TiO_2_ (before adsorption) and C/TiO_2_ + RB-5 (after adsorption). Operating conditions: X-ray source (Al Kα = 1486.7 eV); power: 280 W; energy step: 50 eV; fitting: Gaussian–Lorentzian function.

The XPS analysis was carried out to complement the structural characteristics of the composite and to achieve a more comprehensive understanding of the surface states of the prepared C/TiO_2_ composite, before and after adsorption (Figures 4 and S2). In Figure S2, it is possible to observe a peak around 460 eV, which can be assigned to the chemical Ti–C bonding.

The quantification of the species present in the survey and high-resolution regions for carbon (C 1s) can be better examined in Table 3.

**Table 3:** XPS Analysis Data for the Survey and the C 1s Region

	survey	
	C/TiO_2_ (%)	C/TiO_2_ + RB-5 (%)
O 1s	69.31	67.81
C 1s	23.79	23.28
Ti 2p	6.90	6.75
N 1s	0.00	0.65
S 2p	0.00	1.51

C 1s (high resolution)		
C Sp2	61.44%	49.84%
C Sp3	18.04%	15.09%
C–OH	11.15%	12.25%
C–O	0.05%	6.50%
C=O	9.32%	16.32%

The survey analysis (Figure 4A) of C/TiO_2_ before adsorption revealed a surface predominantly composed of oxygen and carbon, with mass proportions of 69.31% and 23.79%, respectively, in addition to the presence of titanium from the anatase structure, with 6.90%. After the adsorption of Reactive Black 5 (RB-5) dye, the levels of oxygen and, especially, carbon decreased to 67.81% and 23.28%, respectively. According to Khaniya et al.,^74^ this behavior suggests that mechanical stress during the adsorption process causes wear on the C/TiO_2_ coating, reducing the presence of lamellar carbon on the structure after adsorption. Moreover, sulfur and nitrogen were detected on the surface of the material after the adsorption process, with mass proportions of 0.67% (N) and 1.51% (S). This confirms the effective presence of RB-5 adsorbed to the carbon coating on the C/TiO_2_ composite. Regarding the high-resolution C 1s spectra (Figure 4B), decreases in the Sp^2^ and Sp^3^ subcomponents were observed after RB-5 adsorption. As explained by the survey data, this may be attributed to the loss of amorphous carbon coating during the adsorption batches. Furthermore, it was found that the C=O and C–O subcomponents showed a significant increase after the adsorption process. These changes suggest the formation of new chemical bonds between the RB-5 dye and the surface of the composite, reinforcing the hypothesis that the adsorption process is closely related to the direct interaction between the dye and the active sites present on the material surface. The high-resolution spectra for O 1s and Ti 2p also show certain differences before and after adsorption, specifically in the C–O subcomponent (O 1s) (Figure S2). These spectra reveal a slight increase in the percentage of C–O and C–OH bonds, which reaffirms the propensity for new bonds to form between the dye molecule and the composite.^75^

The literature suggests that surface analysis through XPS is a valuable qualitative and quantitative tool for monitoring processes that occur on the surface rather than in the bulk. In a similar study, Foroutan et al.^76^ characterized a magnetic orange seed/Fe_3_O_4_ composite for the adsorption of crystal violet dye. The authors observed higher oxygen content after adsorption (survey data) and an increase in C–O and C–C bonded structures (C 1s), indicating effective dye binding to the composite surface, which is consistent with the findings of the present study.

#### 3.1.5 Determination of Area and Porosity by N_2_ Physisorption

N_2_ physisorption isotherms at low temperatures can validate the intrinsic characteristics of the area and porosity of adsorbent materials. According to Brunauer, Emmet & Teller (1938), porous materials are often characterized by their pore size and distribution, which are obtained through data from gas sorption isotherms. Moreover, the International Union of Pure and Applied Chemistry (IUPAC) proposes classifying isotherms by the relationship between porosity and gas sorption.^78^ The results revealed an isothermic profile consistent with the IUPAC Type-II model, as demonstrated in the isotherm plot (Figure S3). This type of isotherm has a logarithmic profile (a relative concave *p*/*p*° pressure range), whereas its adsorbed quantity approaches a limiting value at the *p*/*p*° = 1.0 threshold. Thus, this type of isotherm describes adsorption limited to a few overlapping adsorbate layers. These coating conditions have often been validated for chemisorption processes as the asymptotic behavior of the adsorption isotherm and the rapid stabilization of the charge saturates the sites that are suitable for electron exchange or sharing reactions.^77,79^

Regarding N_2_ physisorption in the composite, type II isotherms predominantly consist of mesoporous materials.^80^ Thus, filling these structures with thick coatings can only occur under low relative pressures. This trend consists of high affinity between the adsorbate (N_2_) and the adsorbent (composite) and the accessible area in the mesopore structure. The favorable interaction between the solid and gaseous N_2_ enables adsorption onto the material to follow the pore volume rather than the internal surface.^81^

The observed hysteresis in the behavior of the desorption can be classified as type H3, following the nomenclature established by the Union of Pure and Applied Chemistry (IUPAC). As a result of this configuration, hysteresis shows no significant limitations in adsorption at high relative pressures (*p*/*p*°), indicating an energy difference between evaporation and condensation in the innermost pore structures.^80^ These characteristics of the porous structure of the material stem from rigid aggregates resembling plates that probably result in crack-shaped pores. This porous profile directly influences how impediment affects site filling.^82^ Moreover, as it occurs in the H3 pattern, the sorption loop suggests a sudden variation (slope) in relative pressure from 0.4 to 0.45 due to the effects of the steric stress the N_2_ molecules undergo during capillary condensation.^83^Table 4 shows the complete list of the parameters obtained through the area and porosity analysis.

**Table 4:** Parameters from the Area and Porosity Analysis of the C/TiO_2_ Composite Based on N_2_ Physisorption Tests^a^

N_2_ physisorption	
BET surface area (m^2^ g^–1^)	56.24
Langmuir surface area (m^2^ g^–1^)	58.50
area by *t*-plot method (m^2^ g^–1^)	34.31
pore total volume (cm^3^ g^–1^)	0.128
frequent pore diameter (nm)	2.150
average pore diameter (nm)	9.410

a Experimental conditions: *p*/*p*° range = 0.000–0.999; method: mesopore analysis; time = 4.5 h; adsorbate/adsorptive = N_2_ (g); temperature = 77.2 K.

The data in Table 4 show that the composite has a surface area of 56.24 m^2^ g^–1^ according to the BET method and of 58.50 m^2^ g^–1^ according to the Langmuir equation. The literature contains similar BET area values for ZnFe_2_O_4_@Chitosan^84^ and PFSA/SiO_2_-based composites^85^ of around 45.68 and 56.25 m^2^ g^–1^, respectively. Moreover, the C/TiO_2_ composite shows promising areas for pesticide removal in comparison to synthetic adsorbents such as those based on the metal–organic framework HKUST-1@SiO_2_, with areas of around 12.5–18.3 m^2^ g^–1^.^86^ Regarding total pore volume, this study found that C/TiO_2_ showed values of around 0.128 cm^3^ g^–1^, which is in agreement with Ruan et al.^87^ Additionally, the Barrett, Joyner, and Halenda adsorption model validated a frequent pore diameter of around 2.150 nm, categorizing the composite as a material with a predominantly mesoporous structure.^88^ Thus, the pore distribution profile in C/TiO_2_ showed that about 85.41% of the total pore size was in the distribution ranges from 2.05 to 20.0 nm.

#### 3.1.6 pH_PZC_ Estimation of the Adsorbent Material

pH is a highly important parameter that directly impacts the adsorption of a contaminant by an adsorbent. Thus, the intensity of the effect of the pH of the medium can lead to different degrees of removal as it determines the distribution of the chemical species in solution and the surface charge of the adsorbent. The pH at the point of zero charge (pH_PZC_) is an intrinsic characteristic of the surface of solid materials, being strongly influenced by several factors, including the chemical nature of the surface, material composition, and environmental conditions. Figure S4 shows the ΔpH (pH_final_ – pH_initial_) versus pH_final_, which corresponds to a sigmoid curve, the intersection of which with the *x* axis corresponds to the pH_PZC_.^89^ In addition to the sigmoid curve of ΔpH in relation to pH_f_, Figure S4 also shows the graph of pH_0_ versus pH_f_ (insertion).

The conducted analysis shows that the pH_PZC_ of the adsorbent material equals 7.47. Under experimental conditions at which pH_solution_ < pH_PZC_, the surface of the adsorbent material is positively charged, favoring interaction with anionic molecules. On the other hand, at pH _solution_ > pH_PZC,_ the surface of the adsorbent will predominantly show negative charges, favoring the adsorption of species with a positive net charge.^51,89^ The working pH range of the C/TiO_2_ composite (pH = 5.50) should favor RB-5 adsorption since this anionic dye has a greater propensity to adsorb onto positively charged surfaces.^52^

### 3.2 Effect of pH on Adsorption

**Figure 5:**
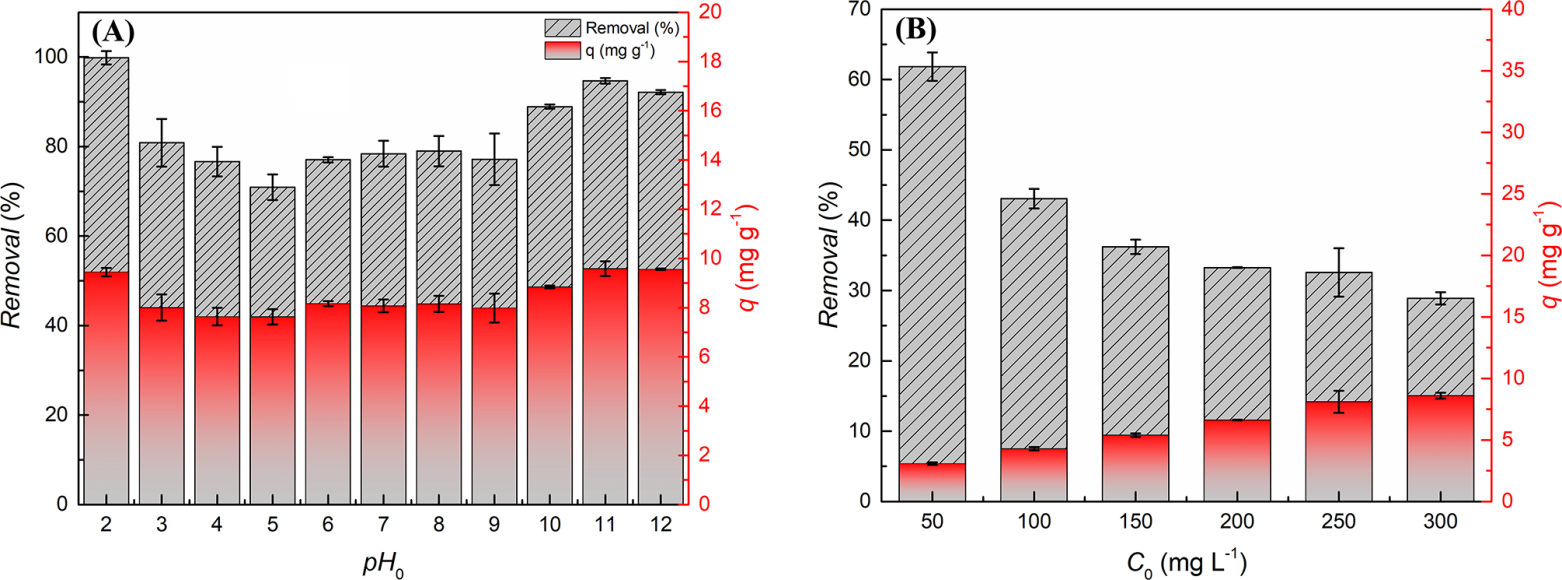
(A) Effect of initial pH on removal percentage (%) and loading (*q*) in the adsorption of RB-5 by the C/TiO_2_ composite. Experimental conditions: *t* = 1440 min, *T* = 25 °C, pH_0_ = 2–12, dosage: 10 g L^–1^; agitation: 100 rpm, [RB-5]_0_ = 50 mg L^–1^. (B) Removal percentage (%) and loading (*q*) profiles related to the effect of initial concentration on RB-5 adsorption in the C/TiO_2_ composite. Experimental conditions: *t* = 1440 min; *T* = 25 °C; [RB-5]_0_ = 50; 100; 150; 200; 250 and 300 mg L^–1^; dosage: 10 g L^–1^; agitation: 100 rpm.

Figure 5A shows the general behavior of adsorption according to variations in the initial pH of the medium. This figure shows the dye removal and loading behavior at different pH values.

The test obtained two behavior patterns, the first of which refers to a significant increase in capacities and loads at more extreme pH conditions and the second, to the constancy in removal parameters at intermediate pH conditions. At pH conditions from 2.0 to 3.0, the surface of the adsorbent material is preferentially charged with positive charges due to the abundant concentration of protons in the medium. The anionic character of RB-5 has a higher affinity with positively charged surfaces and shows the most accentuated removal and loading parameters in this pH range.^52^ On the other hand, high removal percentages also occurred for more alkaline pH ranges, which can be attributed to the low stability of RB-5 under very alkaline pH conditions. Thus, the high values of dye removal are largely attributed to degradation in the range from 10.0 to 12.0.^90^

A second behavior pattern is related to the almost constant removal and loading in the pH range from 4.0 to 9.0, in which average removal equals about 79.4% and loading, 8.00 mg g^–1^. In view of these behavior patterns, the optimal pH range for the adsorption tests will vary from 5.0 to 8.0 since (*i*) the pH of systems with real effluents contaminated by dyes range from 5.0 to 9.0; and (*ii*) adjusting the pH before treating effluents is economically expensive.^50,91^ Therefore, in the subsequent batches, the pH for the development of the experiments will total 5.50.

### 3.3 Effect of Initial Concentration

The methodology described evaluated the behavior of the adsorption process according to variations in the initial concentration of the adsorbate. Thus, Figure 5B shows the removal and loading profiles related to RB-5 adsorption by C/TiO_2_. The graph shows that removal percentages and loading *q* (mg g^–1^) are intrinsically linked to the initial concentration of the solution. As the solution concentration increases, the number of adsorptive species and the demand for active adsorption sites also rises. At high concentrations, the large number of adsorptive molecules in solution limits the number of sites available for adsorption. Thus, removal obtains low values since the surface saturates more easily. However, at low concentrations, the number of potential adsorption sites is sufficiently large when compared to the number of adsorptive molecules, so the surface is not easily saturated. On the other hand, increasing loading concentration has the opposite effect to that of removal^50,91^ as loading increases with a higher initial concentration of the adsorptive.

### 3.4 Determination of the Kinetic Behavior of the Adsorption Process

**Figure 6:**
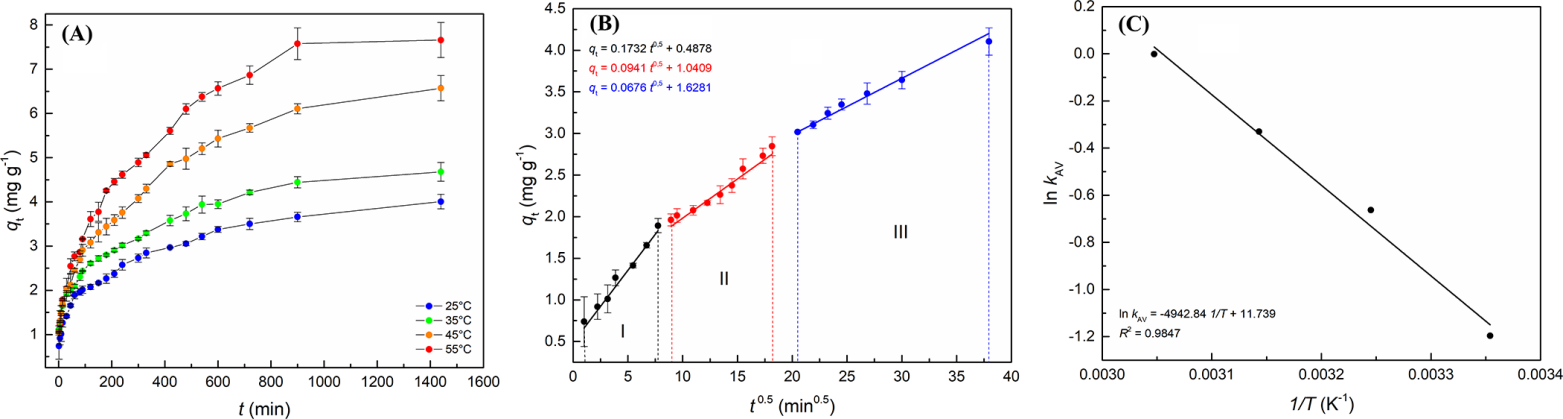
(A) Kinetic behavior of RB-5 adsorption in C/TiO_2_ composite under different temperature conditions. Experimental conditions: *C*_0(RB-5)_ = 100 mg L^–1^; Δ*t* = 1–1440 min; agitation: 100 rpm; Δ*T* = 25–55 °C; adsorbent dosage: 10 g L^–1^. (B) Intrinsic behavior of the multilinear adjustment of the mean experimental data in the Weber and Morris intraparticle diffusion model. Insertion: equations for each line segment/step of the adsorption process. Experimental conditions: *C*_0(RB-5)_ = 100 mg L^–1^; Δ*t* = 1–1440 min; agitation: 100 rpm; *T* = 25 °C; adsorbent dosage: 10 g L^–1^. (C) Linear regression behavior of experimental adsorption kinetics data in the empirical Arrhenius model. Insertion: equation referring to linear regression and coefficient of determination (*R*^2^). Experimental conditions: *C*_0(RB-5)_ = 100 mg L^–1^; Δ*t* = 1–1440 min; agitation: 100 rpm; Δ*T* = 25–55 °C; adsorbent dosage: 10 g L^–1^.

The curves of *q*_*t*_ in relation to *t*, which describe the kinetic adsorption behavior of RB-5 in the composite (Figure 6A), show the effect of temperature variation on adsorption capacity at a time *t*.

Figure 6 illustrates that increasing the temperature significantly raises both the *q*_*t*_ and *q*_e_ values in the threshold region of the kinetic curve. The kinetic studies also facilitated nonlinear regressions (Table 1) of the experimental data using the PFO, PSO, Elovich, and Avrami models across all investigated temperatures (Figure S5). Table 5 presents the kinetic parameters obtained from the regression treatments for each temperature.

**Table 5:** Kinetic Parameters Obtained by Nonlinear Regression Treatments with Experimental PFO, PSO, Elovich, and Avrami Model Data^a^

	25 °C	35 °C	45 °C	55 °C
Pseudo-First Order				
*k*_*1*_ (min^–1^)	0.0112	0.0127	0.0166	0.0058
*q*_e_ (mg g^–1^)	3.174	3.738	5.506	6.803
*R*^2^_adj_	0.7266	0.6205	0.8031	0.8761
χ^*2*^	0.2293	0.4003	0.5105	0.5212
Pseudo-Second Order				
*k*_2_ (g mg^–1^ min^–^1)	0.0046	0.0048	0.0138	0.0009
*q*_e_ (mg g^–1^)	3.542	4.118	6.323	7.951
*R*^2^_adj_	0.8420	0.7645	0.8766	0.9250
χ^*2*^	0.1325	0.2484	0.3199	0.3158
Elovich				
α (mg g^–1^ min^–^1)	0.2131	0.4146	0.1437	0.1213
β (mg g^–^1)	1.6643	1.5656	0.8190	0.6007
*R*^2^_adj_	0.9526	0.9242	0.9451	0.9631
χ^*2*^	0.0397	0.0799	0.1423	0.1550
Avrami				
*k*_AV_ (min^–1^)	0.3023	0.5155	0.7188	0.9985
*q*_e_ (mg g^–1^)	4.954	5.255	6.566	7.852
*n*_AV_	0.2642	0.2426	0.3147	0.4272
*R*^2^_adj_	0.9927	0.9872	0.9892	0.9860
χ^2^	0.6001	0.1372	0.2778	0.5872

a Experimental conditions: *C*_0(RB-5)_ = 100 mg L^–1^; Δ*t* = 1–1440 min; agitation: 100 rpm; Δ*T* = 25–55 °C; adsorbent dosage: 10 g L^–1^.

Table 5 shows that the fractional order in the Avrami model obtained the best regression indicators as its coefficients of determination (*R*^2^) ranged from 0.9927 at 25 °C to 0.9860 at 55 °C. The treatments showed that the Avrami rate (*k*_AV_) and the adsorption capacity at equilibrium (*q*_e_) were highly sensitive to the adopted temperature range. Thus, *k*_AV_ values increased considerably above 30 °C. In addition, theoretical and experimental equilibrium loads varied significantly for the investigated temperature range, whereby the *q*_e,exp_ and *q*_e,teo_ values increased from 4.10 to 4.95 mg g^–1^ at 25 °C and from 7.28 to 7.95 mg g^–1^ at 55 °C, indicating favorable kinetic adsorption at high temperatures. Furthermore, following the Avrami kinetic model predicts that the adsorption process will occur in multiple steps as a single stage fails to determine the overall process. This occurs since the theoretical model has a fractional order of reaction, rather than one in integers (as in the PFO and PSO models). Thus, the Avrami equation suitably describes processes in which the adsorption rate varies over time.^92,93^ The model therefore suggests that Weber and Morris’ considerations explaining mass transfer behavior can be applied to conditions in which the boundary layer effect significantly interferes with adsorption mechanisms.^50^

Based on this assumption, there are several studies in the literature that employ the Avrami model to describe the kinetic behavior of adsorption. Among these studies, Zheng et al.^94^ studied the adsorption of Direct Orange and RB-5 onto a SiO_2_–TiO_2_ polymeric composite. Working concentrations totaled 250 mg L^–1^ for direct orange and 100 mg L^–1^ for RB-5 at pH = 6.5, a temperature of 25 °C, and contact time of 720 min. Results showed an Avrami-calculated loading of 6.988 mg g^–1^ at 25 °C and of 12.65 mg g^–1^ at 55 °C. The kinetic constants also followed a general upward trend, corroborating the kinetic favor of the processes at high temperatures.

The present study applied the Weber and Morris model in its kinetic treatments to better assess the mass transfer behaviors of the adsorption process and the steps that make up the process. Figure 6B shows the multilinear system resulting from applying the intraparticle diffusion model to the experimental data.

The Weber–Morris model lists a multilinear profile of adsorption basically consisting of three distinct steps. Each step is described by an equation of a straight line, as indicated by Figure 6B, and provides clues for better understanding of mass transfer phenomena. According to the model, the first stage (*i*) corresponds to the diffusion of the adsorbate on the surface of the adsorbent; the second stage (*ii*) is intrinsically linked to the intraparticle diffusion process; and the third stage (*iii*) concerns the system entering equilibrium/steady state.^95^ Adsorption processes in which *C* ≠ 0 show a prevalence of the diffusion resistance mechanism in the film as the higher the *C* value, the greater the effect of the boundary layer on the overall speed of the process. These conditions cause external diffusion that limits the adsorption process. On the other hand, if the constant *C* is null (having a step/straight line with an intersection at its origin), intraparticle diffusion becomes the step that governs the process of mass transfer, in which loading will vary proportionally to the square root of time.^50,92,96^ Based on this, the linear regression resulted in eq 5 for the step corresponding to intraparticle diffusion (Figure 6B, in red)
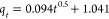
5

Equation 5 shows that the second line failed to directly intersect at its origin (*C* = 1.041 mg g^–1^), demonstrating that intraparticle diffusion fails to fully govern the adsorption process and that several steps are completed during RB-5 removal by the composite.

### 3.5 Effect of Temperature on Adsorption Kinetics

Table 5 shows that the kinetic parameters related to the adsorption process are relatively sensitive to the applied temperature range. The Avrami model showed the best regression indicators. Its *k*_AV_ increased significantly, from 0.3023 (25 °C) to 0.9985 min^–1^ (55 °C), characterizing an increase in adsorption rates with temperature increase. The collected values served to obtain the apparent activation energy of the process according to the Arrhenius empirical model.

Figure 6C shows the linear regression of the values of ln *k*_AV_ as a function of . The equation of the straight line (eq 6)—corresponding to the Arrhenius model—obtained the *E*_A_ of the adsorption process of RB-5 onto C/TiO_2_. In this case, since the slope of the straight line refers to -*E*_A_/*R* and the interception with the *y* axis to ln *A*, this study estimated a value of +41.10 kJ mol^–1^ for *E*_A_ and 1.25 × 10^5^ min^–1^ for the collision factor
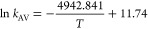
6

In general, the magnitude of energy activation offers insights into the energetic nature of the adsorption process, indicating whether it is predominantly physical or chemical. According to Žunić et al.,^97^ the typical values for *E*_A_ in chemisorption processes lie in the 40–800 kJ mol^–1^ range. On the other hand, lower *E*_A_ values (5–40 kJ mol^–1^) suggest processes predominantly based on physisorption. The value in this study suggests a process governed mainly by activated chemical adsorption. Moreover, the obtained energy level also highlights relatively fast kinetic processes.^98^

### 3.6 Determination of Adsorption Equilibrium

**Figure 7:**
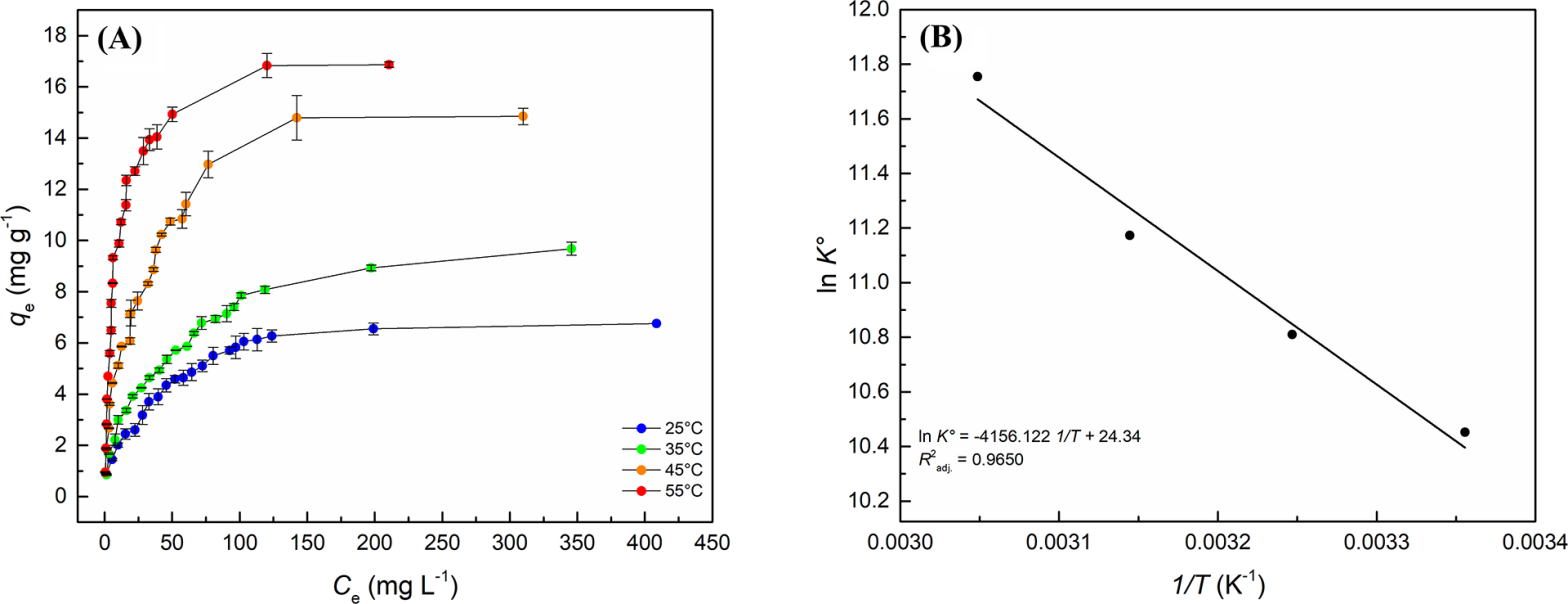
(A) Behavior of experimental RB-5 adsorption equilibrium replicates in C/TiO_2_ under different temperature conditions. Experimental conditions: Δ*C*_0(RB-5)_ = 10 mg L^–1^ at 300 mg L^–1^; *t* = 1440 min; agitation: 100 rpm; Δ*T* = 25–55 °C; adsorbent dosage: 10 g L^–1^. (B) Profile originated from the linear regression of experimental data to the theoretical model of van’t Hoff to obtain the thermodynamic parameters enthalpy and entropy. Experimental conditions: Δ*C*_0(RB-5)_ = 10 mg L^–1^ at 300 mg L^–1^; *t* = 1440 min; agitation: 100 rpm; Δ*T* = 25–55 °C; adsorbent dosage: 100 rpm; Δ*T* = 25–55 °C; adsorbent dosage: 10 g L^–1^.

The adsorption equilibrium experiments obtained the adsorption isotherms from the average experimental values calculated with the replicates. The nonlinear regression treatments of experimental data for the models in Table 2 were able to determine the parameters for each isotherm model. Figure 7A shows the overlaps between the curves of *C*_e_ and *q*_e_ at varying temperatures.

The effect of temperature variation on the system significantly changed the behavior of the loading values in equilibrium, whereby increasing the temperature of the system tends to favor the behavior of the curves. The experimental data were adjusted to the Langmuir, Freundlich, Redlich–Peterson, Sips, Liu, Khan, and Temkin theoretical nonlinear models. Figure S6 shows the individual behaviors of nonlinear regressions at different temperatures. Table 6 shows the parameters obtained by the nonlinear regressions conducted for the curves at 25, 35, 45, and 55 °C.

**Table 6:** Intrinsic Parameters to the Nonlinear Regression of the Mean Equilibrium Experimental Data to the Langmuir, Freundlich, Redlich–Peterson, Sips, Liu, Khan, and Temkin Adsorption Isotherm Models^a^

	25 °C	35 °C	45 °C	55 °C
Langmuir				
*K*_L_ (L mg^–1^)	0.0285	0.0260	0.0416	0.1411
*q*_max_ (mg g^–1^)	7.716	10.40	16.08	17.15
*R*^2^_adj_	0.9758	0.9711	0.9623	0.9902
χ^2^	0.0739	0.1665	0.6006	0.2324
Freundlich				
*K*_F_ (L mg^–1^)	1.271	1.384	2.752	4.548
*n*_F_	3.212	2.828	3.091	3.437
*R*^2^_adj_	0.8888	0.9576	0.9262	0.8780
χ^2^	0.3413	0.2441	1.176	2.897
Redlich–Peterson				
*K*_RP_ (L g^–1^)	0.1986	0.5062	1.060	2.657
*a*_RP_ (mg L^–1^)^−β^	0.0196	0.1415	0.1375	0.1802
β	1.0477	0.8127	0.8659	0.9655
*R*^2^_adj_	0.9755	0.9836	0.9685	0.9909
χ^2^	0.0749	0.0940	0.5016	0.2154
Sips				
*K*_S_ (L mg^–1^)	0.0349	0.0498	0.0717	0.1477
*q*_max_ (mg g^–1^)	7.996	13.71	16.53	17.48
*n*_S_	1.081	1.466	1.405	1.048
*R*^2^_adj_	0.9760	0.9895	0.9781	0.9912
χ^2^	0.0753	0.0604	0.3491	0.2174
Liu				
*K*_Liu_ (L mg^–1^)	0.0264	0.0123	0.0246	0.1345
*q*_max_ (mg g^–1^)	8.003	13.78	19.529	17.46
*n*_L_	0.9229	0.6820	0.7116	0.9536
*R*^2^_adj_	0.9753	0.9890	0.9781	0.9900
χ^2^	0.7588	0.0601	0.3491	0.2374
Khan				
*K*_Khan_ (L mg^–1^)	0.7359	0.7022	0.7423	0.8133
*q*_max_ (mg g^–1^)	1.807	12.55	16.55	17.36
*n*_K_	1.00	1.00	1.00	1.00
*R*^2^_adj_	0.9132	0.9737	0.9538	0.9559
χ^2^	0.2653	0.1511	0.7357	1.046
Temkin				
*a*_T_ (L mol^–1^)	0.6851	0.5883	1.352	2.050
*b*_*t*_ (L g^–1^)	1.923 × 10^3^	1.409 × 10^3^	1.020 × 10^3^	7.781 × 10^2^
*R*^2^_adj_	0.9114	0.9497	0.9175	0.9774
χ^2^	0.2710	0.2898	1.314	0.5360

a Experimental conditions: Δ*C*_0(RB-5)_ = 10 mg L^–1^ at 300 mg L^–1^; *t* = 1440 min; agitation: 100 rpm; Δ*T* = (A) 25 °C. (B) 35 °C. (C) 45 °C and (D) 55 °C; adsorbent dosage: 10 g L^–1^.

Table 6 shows that the Sips isotherm model—followed by the Liu model—was the best fit for the average experimental data, obtaining the highest coefficients of determination, from 0.9760 (Sips) and 0.9756 (Liu) at 25 °C to 0.9912 (Sips) and 0.9900 (Liu) at 55 °C. The Sips model showed that increasing the temperature of the system significantly increased its equilibrium constant (*K*_S_), from 0.0349 L mg^–1^ at 25 °C to 0.1539 L mg^–1^ at 55 °C. Increasing the temperature of the system also improved the maximum adsorption capacity of the material.^99,100^Table 7 comparatively overviews studies on composites to remove textile dyes contaminated by RB-5 that used maximum *q*_e_ values as a general parameter of efficiency.

**Table 7:** Composites and Their Maximum Adsorption Capacities for RB-5 in Aqueous Solution, as Reported in the Literature

adsorbent	*q*_max_ (mg g^–1^)	reference
nGO@Fe_3_O_4_	15.50	(101)
Fe-SA-Y@Fe_3_O_4_	45.45	(102)
ZnO@chitosan	18.94	(99)
SiO_2_–PPY	4.600	(103)
TiO_2_–NPs@biopolymer	10.98	(104)
calcined kaolin/calcium carbonate@TiO_2_	18.90	(105)
Sn–C/TiO_2_	39.10	(106)
TiO_2_@chitosan-montmorillonite	8.900	(107)
cross-linked chitosan-epichlorohydrin@TiO_2_	87.50	(108)
Kaolin@TiO_2_	16.50	(109)
CdS nanocrystals + cross-linked chitosan@TiO_2_	2.660	(110)
cellulose/Chitosan@TiO_2_–NPs	0.606	(111)
grape marc-based activated carbon@TiO_2_	25.00	(112)
activated-carbon-supported titania (ACT)	52.60	(113)
C/TiO_2_	17.48	this work

As these regression models are a theoretical-mathematical approximation between the Langmuir and Freundlich models, the Sips, Liu, and Khan isotherm models predominantly predict chemical adsorption, with the adsorbent material surface with mostly homogeneous sites, variable energy distribution (as per Freundlich), a monolayer coating, and high isosteric enthalpy modules (as in the Langmuir theoretical model).^114^ Additionally, Foo & Hameed^115^ and Chu et al.^116^ show that the Sips model is reduced to the Freundlich isotherm at low absorbate concentrations, whereas the coating regime of the adsorbent solid follows a monolayer system at high concentrations. Moreover, the parameters of the equation mainly follow operating conditions, pH range, temperature, and concentration.^117^

Regarding the other parameters in this study, the Sips coefficient (*n*_S_) shows how the model algebraically and physically approximates to the interpretations of the Langmuir and Freundlich models. This parameter shows values from 1 to 10. If *n*_S_ takes a value closer to 1, the expression of Sips will be purely reduced to the Langmuir equation. On the other hand, if *n*_S_ approaches 10, the adsorption process will be categorized as unfavorable. Furthermore, if *n*_S_ shows intermediate values, the adsorption will follow Freundlich considerations.^114,116,118^ As such, the experimental data showed *n*_S_ values closer to 1.0 for all the investigated temperature conditions, indicating an adsorption process with characteristics close to those described by Langmuir.^119^ Another point to be considered relates to the sensitivity of the Sips equilibrium constants (*K*_s_) to the adopted temperature range. This behavior enables thermodynamic treatments during adsorption. Figure 7B shows the variation of ln *K*_e_° as a function of the inverse temperature.

The obtained regression behavior showed the formation of a straight line with a slope in  and interception with the *y* axis at . The linear equation describing the behavior of the curve follows eq 7
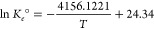
7

Thus, parametrization treatments of the obtained curve can determine the experimental values of Δ_ad_*H*° and Δ_ad_*S*° for the process of dye adsorption onto the composite. Moreover, the Gibbs free energy general equation made it possible to estimate the values of Δ_ad_*G*° for each temperature point. Table 8 shows the thermodynamic parameters calculated for the process.

**Table 8:** Thermodynamic Parameters Obtained from Experimental Data of RB-5 Adsorption onto C/TiO_2_ at Various Temperatures^a^

dye	temperature (K)	*K*_e_°	Δ_ad_*G*° (kJ mol^–1^)	Δ_ad_*H*° (kJ mol^–1^)	Δ_ad_*S*° (J mol^–1^ K^–1^)
RB-5	298	3.46 × 10^4^	–25.90	+34.55	+202.36
	308	4.94 × 10^4^	–27.67		
	318	7.11 × 10^4^	–29.53		
	328	1.46 × 10^5^	–32.43		

a Experimental conditions: Δ*C*_0(RB-5)_ = 10 mg L^–1^ at 300 mg L^–1^; *t* = 1440 min; agitation: 100 rpm; Δ*T* = 25–55 °C; adsorbent dosage: 10 g L^–1^.

The reported thermodynamic data show an endothermic adsorption process with an enthalpy variation of +34.55 kJ mol^–1^. This value suggests that adsorption becomes more favorable as energy is supplied to the system as heat. This is because, considering the adsorption process as an endothermic chemical reaction, the adsorption equilibrium will tend to shift toward the products as the heat flows into the system from its vicinity. Thus, the process will probably be carried out more favorably at higher temperatures.^97,120^

On the other hand, entropy variation can predict the degree of disorder in the system and, as a result, constitutes an important factor in adsorption since entropies above zero reflect the increase in the degrees of freedom of the species in the process. Thus, the movements available to a molecule tend to be greater in the adsorbed state than in the fluid phase, justifying obtaining positive entropy for the process.^50,121^

Another parameter of great importance in adsorption studies is Gibbs free energy. Data show that the values of Δ_ad_*G*° were negative for the entire temperature range, indicating an exergonic, spontaneous, and increasingly favorable process at high temperature conditions. On the other hand, the parameters Δ_ad_*H*° and Δ_ad_*S*° failed to show significant levels of sensitivity to the adopted temperature range, enabling the consideration of their values as practically constant under the investigated conditions.^92,95,122^ Entropic thermodynamic control is attributed to the adsorption process in view of these characteristics since, considering the fundamental Gibbs free energy equation, the entropy term (−*T*Δ*S*) starts to stand out in relation to the positive values of enthalpy, resulting in a negative net variation for Δ*G*.^123^ As a result, the global process becomes increasingly spontaneous and exergonic as *T* increases.

### 3.7 Interaction Effects between the Support, Carbon Layer, and Dye: Exploring a Potential Mechanism for the Adsorption Process

**Figure 8:**
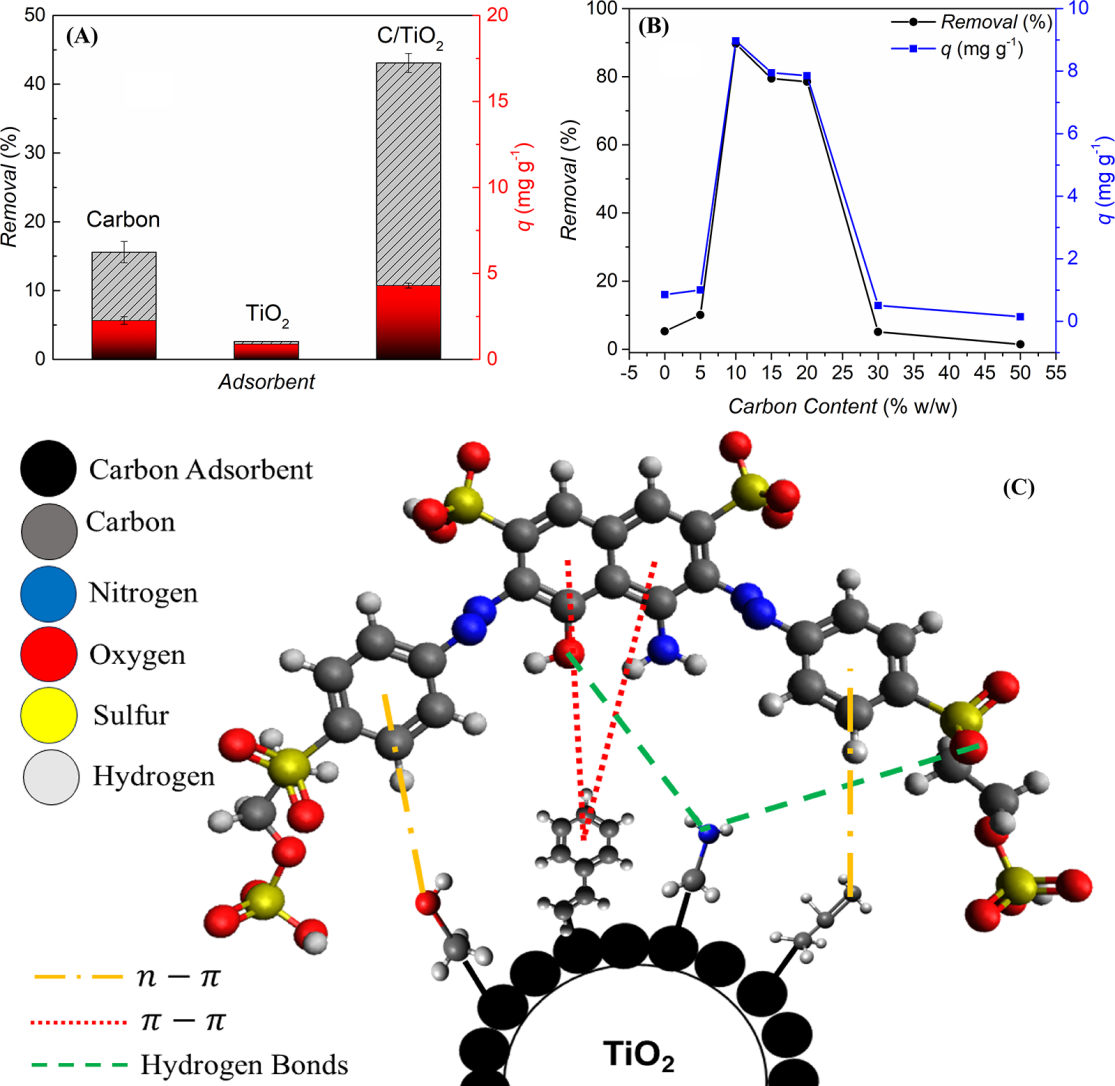
(A) Comparison of the removal percentages and loading capacities of the C/TiO_2_ composite (10% C coating) and its individual components (C and TiO_2_) during the RB-5 removal; (B) relationship between theorical carbon content in the composite and removal percentages and adsorption capacity (*q*) after 180 min of contact of the RB-5 solution with the adsorbent. Operating conditions: *C*_RB-5_ = 50 mg L^–1^, *t* = 180 min, *T* = 25 °C, pH = 5.5, stirring = 100 rpm; dilution: 5×; (C) schematic diagram for possible mechanisms involved in the adsorption process of RB-5 onto C/TiO_2_ composite.

After considering the kinetic and thermodynamic parameters for the adsorption process, it is necessary to investigate the main characteristics and a possible mechanism that explains the process of dye adsorption onto the composite both macroscopically and as an interaction. Macroscopically, adsorption experiments using the individual components of C/TiO_2_, and varying carbon cover contents were conducted to evaluate the influence of composite formation on removal efficiency. Figure 8A compares the adsorption capacities of the C/TiO_2_ composite (10% C coating) with those of its individual components (C and TiO_2_) at an initial dye concentration of 100 mg L^–1^.

Comparative adsorption studies using the individual components of the C/TiO_2_ composite (isolated C and TiO_2_) were conducted in the present investigation to elucidate the effect of combining these species (composite formation) on the overall efficiency of the adsorbent for RB-5 removal. In this regard, Figure 8A shows the removal percentages and subsequent loadings reported for RB-5 removal using different adsorbents. Thus, the most efficient adsorption process was observed in experiments conducted with the C/TiO_2_ composite, achieving a loading of 4.30 mg g^–1^ and a removal efficiency of 43.08% over a total experimental time of 180 min. On the other hand, experiments conducted with carbon derived from the pyrolysis of sucrose showed modest removal, reaching values of 15.58% and a loading of 2.25 mg g^–1^, while isolated TiO_2_ exhibited virtually zero adsorption efficiency. These findings indicate that the formation of the composite enhanced the efficiency of the process, as the stabilizing effect of the TiO_2_ support on the carbon coating facilitated the formation of stronger, lower-energy surface-molecule bonds, as highlighted by Huo et al.^124^ Additionally, Liu et al.^47^ emphasize that the hydrophobic nature of TiO_2_ can greatly improve the conformational stability of carbon structures and the adsorption capacity of pollutants due to π–π electron donor–acceptor interactions and pore-filling effects. The combined effects result in significant improvements to the final material when compared to the separate application of its individual components.

Figure 8B illustrates the relationship between theoretical carbon content and dye removal efficiency/loading of the C/TiO_2_ composite. In addition, the experimental carbon content, determined through gravimetric analysis in a muffle furnace, along with its correspondence to the theoretical content, is presented in Table S3. In this context, composites with theoretical carbon contents of 10% (exp. 9.23%), 15% (exp. 13.16%), and 20% (exp. 23.48%) demonstrated the highest dye removal efficiencies in synthetic effluent, reaching 89.66%, 79.48%, and 78.53%, respectively. On the other hand, theoretical compositions of 0% and 50% (exp. 50.41%) showed the lowest removal rates, approximately 5.25% and 4.56%, respectively. According to Huo et al.,^124^ this characteristic indicates the impact of the degree of carbon coating on the surface of the TiO_2_ in supporting the adsorption process.^124^ As a result, the efficiency of the composite decreases with increased coatings since an excessive amount of carbon can obstruct the pores in the innermost layers, making it difficult for molecules to access these regions, thus impairing adsorption.^121,125^ Moreover, with the absence of or low carbon content, as in 0% C and 5% C (exp. 4.57%), the scarcity of carbonaceous structures may contribute to two effects: (*i*) a reduction in the availability of functional groups able to interact with the dye molecule groups; and (*ii*) a substantial reduction in surface area due to the decrease in the rough/porous structure of the surface.^121^ Both factors decrease the percentage of dye removal in aqueous solutions.

Regarding the explanation of a possible mechanism for surface-molecule interactions, different types of bonds may be involved during adsorption. Specifically, hydrogen bond-based interactions, π–π (pi stacking), *n*–π, and electrostatic forces probably occur in the process due to the presence and availability of certain functional groups in C/TiO_2_ and RB-5.^126^ Therefore, Figure 8C shows a probable interactional profile between RB-5 and C/TiO_2_. The diagram shows that the structure of the dye molecule contains the groups –N=N– (azo) and [R–S(=O)_2_] (sulfone), bonds between two or more aromatic rings, and the –OH (hydroxyl) and –NH_2_ (amine) groups, whereas the composite showed hydroxyl, amine, aliphatic alkene, and alkenes linked to aromatic groups (according to Fourier transform infrared spectroscopy), which can act as active sites in chemical adsorption processes. Considering these characteristics, the hydroxyl groups on the surface of the adsorbent can establish two distinct modes of interaction: (*i*) the formation of hydrogen bonds between nitrogen and oxygen atoms in the dye; or (*ii*) the interaction of the same hydroxyl group in C/TiO_2_ with the aromatic ring of RB-5 to promote *n*–π bonds.^127^ In the case of the latter, hydroxyl groups would play the role of electron donors (nucleophiles), interacting with electron-rich π regions (π-acceptors) in the RB-5 molecule as aromatic rings. This bond would considerably stabilize the adsorbate–adsorbent system, promoting greater fixation on its surface.^128,129^ It is also important to highlight that –NH_2_ can establish hydrogen bonds with the oxygen atoms in the sulfone group and the –OH groups attached to the aromatic ring of RB-5.

Furthermore, the carbon coating on the surface of the composite contains alkenes and binds to aromatic rings, which enables pi-stacking (π–π) bonds between the dye and the surface. These interactions contain noncovalent attractive forces between two overlapping aromatic rings in spatial conformation *T*, ensuring a high degree of coupling and a stable final bond.^130^ Based on this assumption, the aromatic rings of the dye molecule can interact with the rings in the composite coating, better approximating/fixating the dye to the C/TiO_2_.^131,132^

Finally, the results showed that the C/TiO_2_ composite is a promising material for application in the purification of textile effluents due to its good adsorption capacity and good cost-efficiency ratio. Additionally, as TiO_2_ is able to generate hydroxyl radicals through photocatalytic reactions, future studies considering adsorption followed by photodegradation of pollutants are in progress.

## 4 Conclusion

The development of new materials has become a strong need for modern societies. Thus, preparing materials for adsorption is essential for developing more sustainable and environmentally appropriate purification methodologies. Therefore, this study sought to synthesize a C/TiO_2_-based composite to remove RB-5 textile dye in aqueous media. Previous tests showed an adequate pH range from 5.0 to 8.0 for the adsorption tests since the pH values of actual effluents lie within this range. The effect of initial concentrations also showed that the optimal concentration range lies from 10 to 300 mg L^–1^. The RB-5 adsorption process onto the composite followed an Avrami fractional kinetic model, with a rate totaling 0.9985 min^–1^ and a maximum load at equilibrium equal to 7.95 mg g^–1^ at a temperature of 55 °C. Additionally, the process can be described by the multiple steps governing adsorption, in which no single stage determines the overall process. Thus, the Weber–Morris model was successfully applied to the experimental data, showing that adsorption occurred in three stages, and that all linear stages pertinent to the interpolation of the experimental data failed to directly intercept the origin, demonstrating preferential adsorption onto the outer surface of the composite. The activation energy apparent to adsorption totaled +41.10 kJ mol^–1^, supporting chemisorption. The equilibrium analyses validated a Sips adsorption model, with a maximum loading of 17.48 mg g^–1^ at 55 °C. Regarding the parameters of the model, *n*_S_ approached 1.0, indicating adsorption based on a coating of chemically adsorbed monolayers. Thermodynamic analysis demonstrated an endothermic chemical process (Δ*H* = +34,55 kJ mol^–1^) followed by an increase in the degrees of freedom of the target molecule and increasingly exergonic free energy values with a rise in temperature. A possible mechanism to explain the surface–dye interaction process suggests that adsorption follows hydrogen bond, π–π, and *n*–π interactions, whereas the compositional control over the carbon content in the coating is fundamental to promoting more effective processes. Thus, the C/TiO_2_ composite provides a sustainable, viable, and promising alternative for the removal of RB-5 from aqueous solutions, showing great potential to decontaminate effluents.

## Supporting Information Available

SEM micrographs related to C/TiO_2_ under conditions before and after the RB-5 adsorption process; XPS high-resolution spectra for O 1s and Ti 2p; plots of nitrogen adsorption–desorption isotherms at low temperatures, along with pore size distribution via the BJH method; a graph of final pH (pH_f_) versus ΔpH for determining the point of zero charge (pH_pzc_); nonlinear regression behavior of adsorption kinetic curves for the PFO, PSO, Elovich, and Avrami models at different temperatures; and nonlinear regression behavior of adsorption equilibrium data for the theoretical models of Langmuir, Freundlich, Redlich–Peterson, Sips, Liu, Khan, and Temkin at various temperatures. In addition to the figures indexed as Supporting Information, the PDF contains Table S1, which presents the elemental analysis and EDS spectra quantification; Table S2, which covers the parameters from Raman spectroscopy analysis (D, G, and 2D peaks), as well as the intensity ratios *I*_D_/*I*_G_; and Table S3, which demonstrates the relationship between the nominal and experimental carbon content determined through gravimetric analysis in a muffle furnace (PDF)
