# Supplementary material for: Synthesis, Characterization, and Application of an Ecofriendly C/TiO2 Composite to Efficiently Remove Reactive Black 5 (RB-5) Textile Dye from Aqueous Solutions
Source: ACS Omega. 2025 Mar 18;10(12):12241–59. doi: 10.1021/acsomega.4c10884 (PMC11966260; doi:10.1021/acsomega.4c10884)
Supplement: Supplementary file 1 — ao4c10884_si_001.pdf [file ao4c10884_si_001.pdf]

Supplementary Materials

Synthesis, Characterization, and Application of an Ecofriendly C/TiO_2 Composite to Efficiently Remove Reactive Black 5 (RB-5) Textile Dye from Aqueous Solutions

Lucas Destefani Paquini[a], Lília Togneri Marconsini[b], Bruno Sanches de Lima[c], Luciene Paula Roberto Profeti[b], Josimar Ribeiro[a], Demetrius Profeti[b]*

[a] Laboratório de Pesquisa e Desenvolvimento em Eletroquímica (LPDE), Universidade Federal do Espírito Santo, Campus Goiabeiras, Av. Fernando Ferrari, 29075-910 Vitória, ES, Brazil

[b] Programa de Pós-Graduação em Agroquímica, Universidade Federal do Espírito Santo, Alto Universitário, s/n., 29500-000 Alegre, ES, Brazil

[c] Instituto de Física Gleb Wataghin, Universidade Estadual de Campinas - UNICAMP, 13083-859 Campinas, SP, Brazil

KEYWORDS: Effluents. Dyes. Adsorption. Composites. Efficiency.

*** Corresponding author**

Name: Demetrius Profeti

Telephone: +55 (28) 3552-8669

E-mail: demetrius.profeti@ufes.br

The PDF file includes:

Figure S1. Micrographs of the C/TiO_2 composite obtained by Scanning Electron Microscopy.
Figure S2. High-resolution XPS spectra for O 1s and Ti 2p.
Figure S3. Profile related to physical adsorption and desorption of N_2 isotherms at 77.2 K onto C/TiO_2 composite.
Figure S4. Graphics of pH variation as a function of final pH (initial pH versus final pH) to determinate the pH_{ZCP} of the C/TiO_2 composite.
Figure S5. Nonlinear regression curves of the kinetics experimental data to the theoretical models of PPO, PSO, Elovich, and Avrami for fractional order.
Figure S6. Nonlinear regression curves of the equilibrium experimental data to the theoretical models of Langmuir, Freundlich, Redlich-Peterson, Sips, Liu, Khan, and Temkin.
Table S1. Elementary analysis data of the C/TiO_2 composite obtained by Energy Dispersive Spectroscopy (EDS).
Table S2. Summary report of Raman spectroscopy analysis.
Table S3. Determination of the experimental carbon content in the coating of the C/TiO_2 composite.

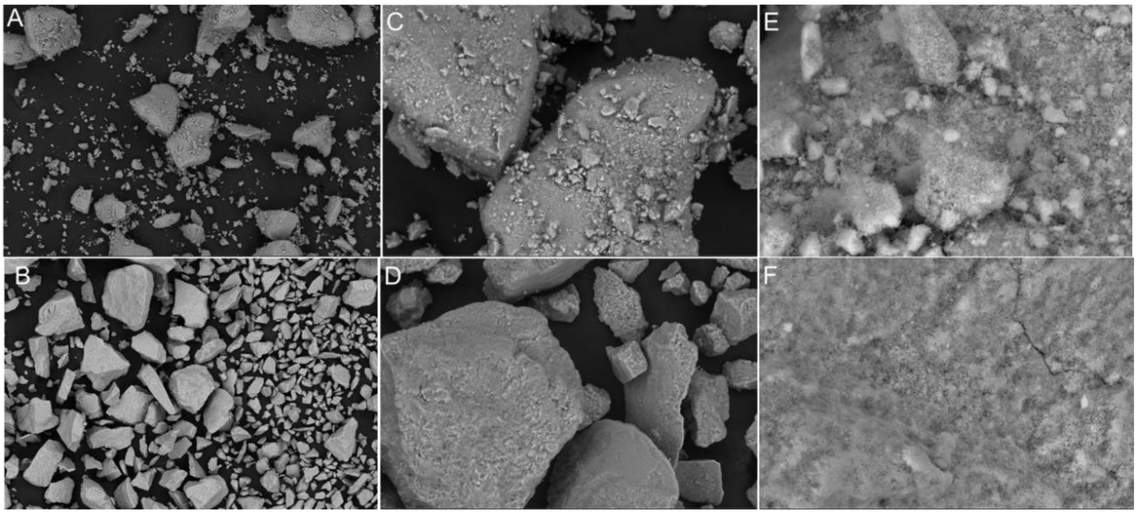

Figure S1. Micrographs of the C/TiO_2 composite obtained by Scanning Electron Microscopy, under conditions before (A, C, and E) and after (B, D, and F) adsorption with RB-5. Magnifications: (A) and (B) 100x; (C) and (D) 1000x; (E) and (F) 2500x.

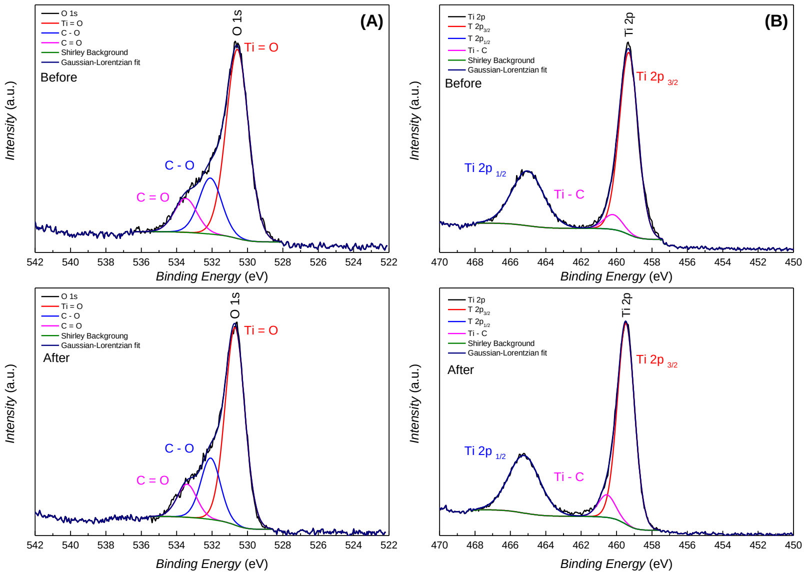

Figure S2. XPS High-resolution spectral profiles for the (A) O 1s analysis (components: Ti = O, C – O, and C = O) and (B) Ti 2p analysis (components: Ti 2p$_{3/2}$, Ti 2p$_{1/2}$ and Ti-C) before and after the adsorption experiment with RB-5 azo dye.

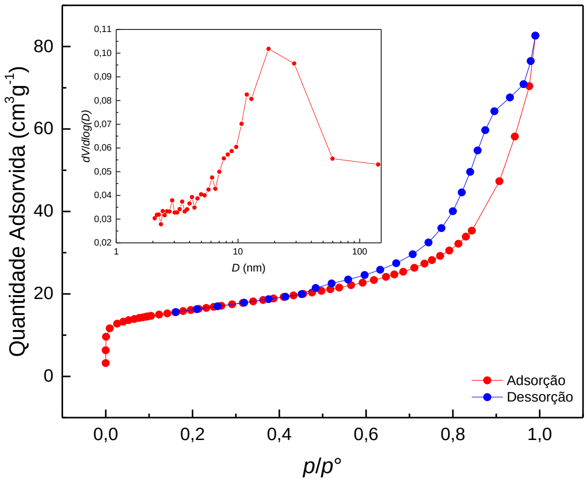

Figure S3. Profile related to adsorption (-•-) and desorption (-•-) of N$_2$ gaseous isotherms at 77.2 K on the C/TiO$_2$ composite. Insertion: Pore distribution profile following the Barrett, Joyner, and Halenda model for N$_2$ adsorption data. Experimental conditions: w/w° range = 0.000-0.999; Method = mesopore analysis; time = 4.5 h; Adsorptive/Adsorbed = N$_2$(g); T = 77.2 K.

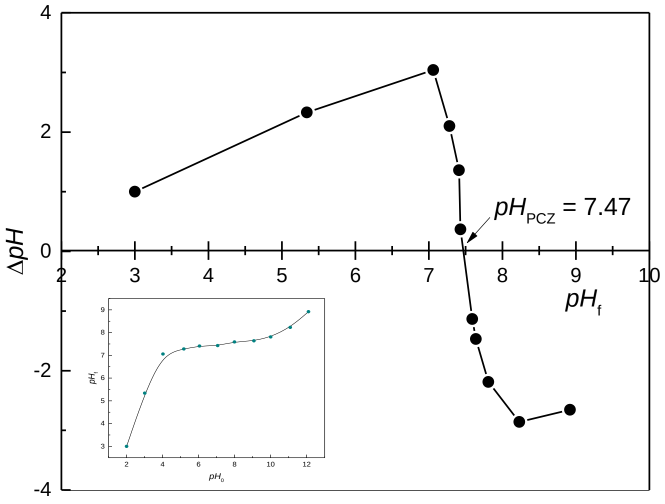

Figure S4. Graphics of pH variation as a function of final pH (initial pH versus final pH) to determinate the pH_{ZCP} of the C/TiO_2 composite. Experimental conditions: t = 1440 min, T = 25 °C, dosage: 10 g L^{-1}, pH range = 2 – 12.

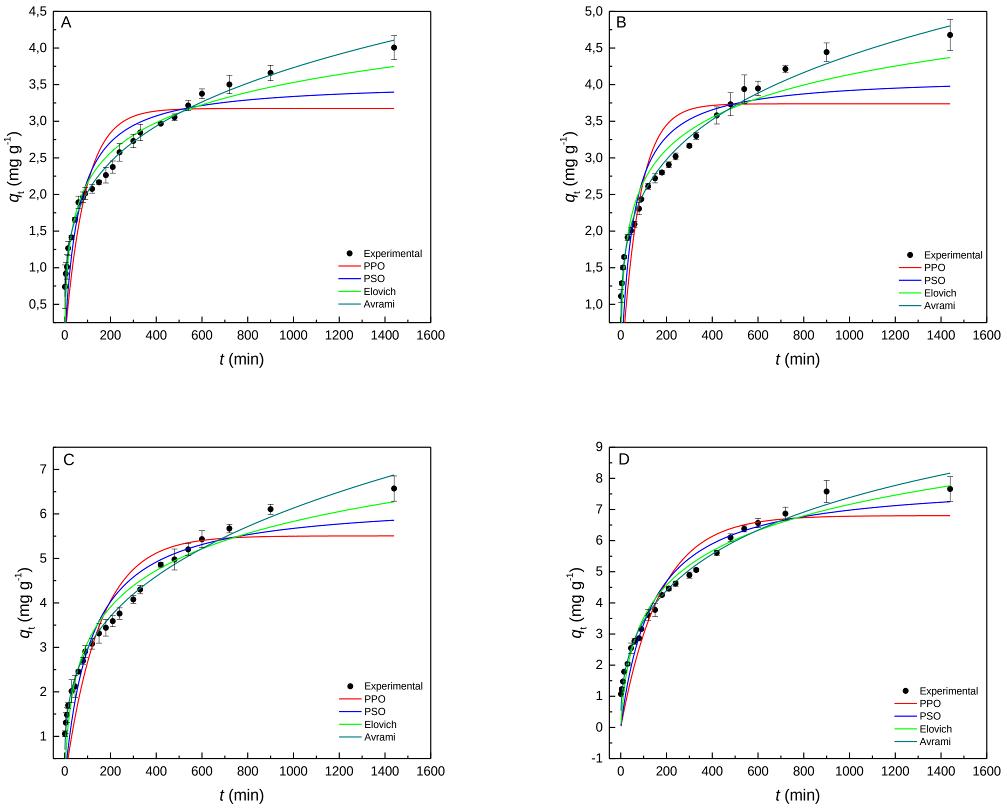

Figure S5. Nonlinear regression curves of the average experimental data to the theoretical models of PFO, PSO, Elovich, and Avrami for fractional order. Experimental conditions: C_0

(RB-5) = 100 mg L⁻¹; Δt = 1 – 1440 min; agitation: 100 rpm; ΔT = (A) 25°C, (B) 35°C, (C) 45°C, and (D) 55°C; adsorbent dosage: 10 g L^{-1}.

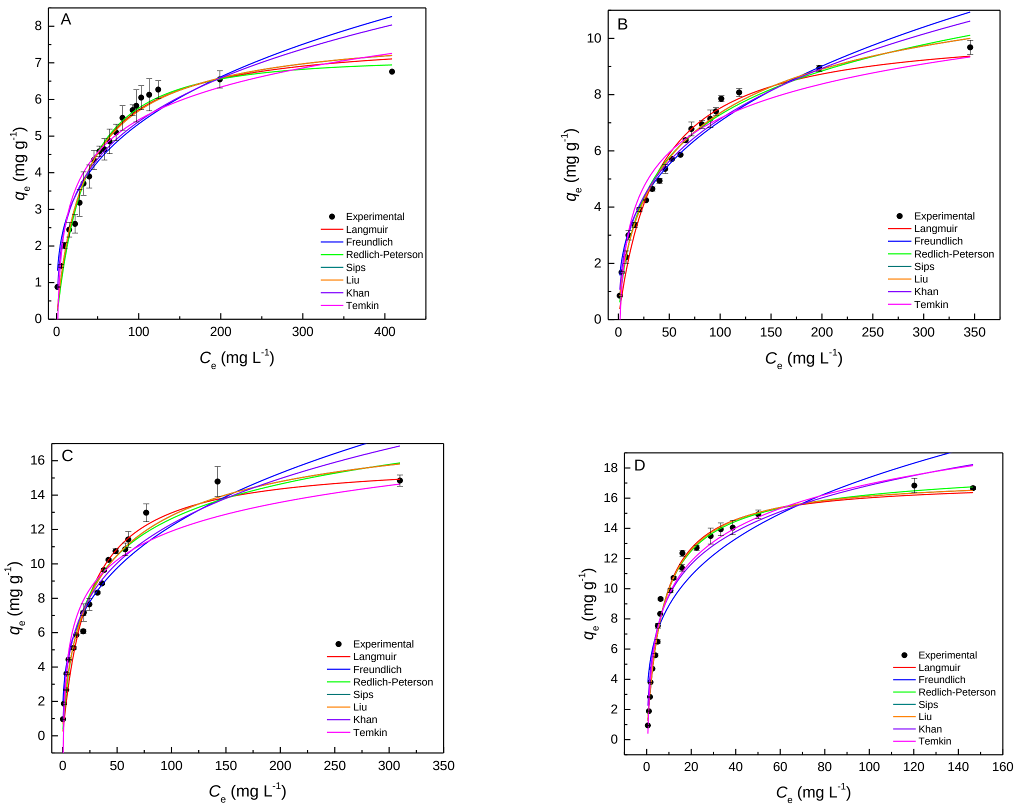

Figure S6. Nonlinear regression curves of the average experimental data to the theoretical models of Langmuir, Freundlich, Redlich-Peterson, Sips, Liu, Khan, and Temkin. Experimental conditions: ΔC_0 (RB-5) = 10 mg L⁻¹ to 300 mg L^{-1}; t = 1440 min; agitation: 100 rpm; ΔT = (A) 25°C, (B) 35°C, (C) 45°C, and (D) 55°C; adsorbent dosage: 10 g L^{-1}.

Table S1. Elementary analysis data of the C/TiO_2 composite obtained by Energy Dispersive Spectroscopy (EDS), before and after the adsorption process of the RB-5 dye.

Element	Before adsorption (%wt.)	After adsorption (%wt.)
O	60.66	22.3
Ti	19.2	58.6
C	20.14	19.1
Total	100.00	100.00

Table S2. Summary of Raman spectroscopy analysis.

Material	D	G	2D	I_D/I_G
C/TiO_2	1352.54	1589.68	2774.38	1,065
C/TiO_2 + RB-5	1358.37	1593.26	2804.44	1,057

Table S3. Determination of the experimental carbon content in the coating of the C/TiO_2 composite, performed through gravimetric analysis in a muffle furnace.

Weight	Nominal Content					
	5%	10%	15%	20%	30%	50%
Before Calcination (g)	0.1236	0.1170	0.1056	0.1158	0.1328	0.1198
After Calcination (g)	0.1179	0.1062	0.0916	0.0885	0.0895	0.0594
Carbon Weight (g)	0.00565	0.0108	0.0139	0.0272	0.0432	0.0604
**Experimental Content (%)**	**4.57 %**	**9.23 %**	**13.16 %**	**23.48 %**	**32.53 %**	**50.41 %**
